# What to Say When It Matters: Communication Skills to Address Implicit Bias Workshop

**DOI:** 10.15766/mep_2374-8265.11514

**Published:** 2025-04-15

**Authors:** Bria McKenzie, Dhanya Mahesh, Elena Gupta, Phuong Pham, Adina Harri, Erin M. Knight, Bich T. N. Tran, Arvind Suresh, Maya Ellis, Nan Cochran, Alison Kapadia

**Affiliations:** 1 Third-Year Medical Student, Geisel School of Medicine at Dartmouth; 2 Fourth-Year Medical Student, Geisel School of Medicine at Dartmouth; 3 Second-Year Medical Student, Geisel School of Medicine at Dartmouth; 4 Research Scientist and Senior Research Associate, Center for Program Development and Evaluation and The Dartmouth Institute for Health Policy and Clinical Practice, Geisel School of Medicine at Dartmouth; 5 Research and Evaluation Associate, Center for Program Development and Evaluation and The Dartmouth Institute for Health Policy and Clinical Practice, Geisel School of Medicine at Dartmouth; 6 Second-Year Resident, Department of Medicine, University of California, San Francisco, School of Medicine; 7 First-Year Resident, Department of Family Medicine, Contra Costa Health; 8 Associate Professor, Department of Medicine, The Dartmouth Institute for Health Policy and Clinical Practice, Geisel School of Medicine at Dartmouth; 9 Assistant Professor, Department of Emergency Medicine, Dartmouth Health and Geisel School of Medicine at Dartmouth

**Keywords:** Implicit Bias, Interdisciplinary Communication, Microaggression, Anti-racism, Bias, Communication Skills, Cultural Competence, Health Equity, Simulation, Diversity, Equity, Inclusion

## Abstract

**Introduction:**

In academic medicine, learners, faculty, and staff commonly experience microaggressions, which have been linked to multiple negative outcomes (e.g., higher levels of depression, deteriorating well-being, increased anxiety, and feelings of isolation). Workshops teaching communication skills can reduce barriers in responding to microaggressions. Our workshops included students, faculty, and staff as both participants and cofacilitators.

**Methods:**

Each 2-hour, in-person workshop began with a large-group didactic. Next, cofacilitators led skills practice responding to microaggressions. Small-group cofacilitators underwent standardized training. Student and faculty/staff participants were surveyed after each workshop. We conducted factorial analyses of variance comparing the main effects of time, role (student vs. staff/faculty), and prior knowledge, as well as the interaction effects between time and role and between time and prior knowledge.

**Results:**

Fourteen workshops were conducted between 2022 and 2023. Students attended four of the workshops, and faculty/staff participated in 10. Participants reported greater knowledge (*p* < .001) and comfort (*p* < .001) identifying and responding to microaggressions after the workshop. The interactions between time and role and between time and prior knowledge on the dependent variables knowledge and comfort were significant (knowledge: *p* < .01; comfort: *p* < .05).

**Discussion:**

Active learning workshops constitute an effective method for teaching communication skills to address implicit bias in academic medicine. Including students as cocreators allows for greater relevance for student participants and authenticity for the student experience. Future directions include examining data on cofacilitator experiences and comparing outcomes for students versus faculty/staff.

## Educational Objectives

By the end of this activity, learners will be able to:

1. Define implicit bias, microaggressions, and stereotype threat.
2. Recognize and constructively respond to microaggressions as a recipient, ally, or source of a microaggression.
3. Increase awareness of their own implicit bias and of actions that may unintentionally engage in subtle acts of exclusion.
4. Develop diverse, equitable, and welcoming medical learning environments characterized by psychological safety and strong allyship.

## Introduction

Subtle acts of exclusion, or microaggressions, are experienced commonly in clinical and learning environments by marginalized groups. Microaggressions may include general disrespect, invalidation, and exclusion of targeted individuals. Examples include assigning a team member responsibilities commonly associated with gender stereotypes or assuming someone's ability is based on their race or accent. Throughout, we refer to subtle acts of exclusion using the common term *microaggressions* coined by Harvard psychiatrist Chester M. Pierce in 1970 to describe insults and dismissals he regularly witnessed non-Black Americans inflicting on African Americans.^1^

Microaggressions are often ignored, and their adverse impact is minimized. However, medical students and residents are particularly likely to experience microaggressions.^2,3^ These experiences cause multiple negative outcomes, such as higher levels of depression and deteriorating well-being,^4^ increased anxiety and feelings of isolation,^5^ and higher rates of considering medical school transfer.^6^ Furthermore, witnessed microaggressions can perpetuate a culture that accepts social norms of exclusion.^7,8^ This decreases a sense of belonging; negatively impacts memory, communication, and performance; and increases burnout.^2^

Current literature includes many models that teach implicit bias recognition and response.^4,9–15^ Many trainings use interactive workshops ranging from 50 to 150 minutes, with time dedicated to practicing the framework.^12,14,16,17^ Common goals include improving self-efficacy, building allyship, and reducing the harm caused by microaggressions in the clinical environment. Implicit bias training initiatives are often requested and driven by students^9^ and generally taught by faculty^12,14,16^ or a senior student with a faculty member.^18–21^

*MedEdPORTAL* has published many studies addressing microaggressions in medical learning environments.^10,12,19,22^ In one instance, authors presented a framework in which small-group participants analyze written cases adapted from student stories and role-play responding to microaggressions. The authors suggested that allowing more workshop time for role-play could enhance students’ practical skills.^10^ Another approach, the VITALS model, incorporates both faculty facilitator-led didactics and small-group peer discussion and skills practice without a faculty facilitator to remove the power dynamic.^12^ This publication recommended extending the length of its workshop in the future.^12^ The 6Ds model, which leverages small-group, case-based learning, involves both near-peer and faculty cofacilitators to “optimize learning climate safety with near-peer instruction.”^19^ Another model utilizes a one-time, small-group, problem-based learning based on student experiences and recommends integrating sessions into a longitudinal curriculum.^22^ In contrast to these earlier publications, our pedagogical approach is unique because it prioritizes extended skills practice to promote proficiency. We also emphasize peer cofacilitation by having medical students, often the same class year as participants, serve as facilitators. Faculty also cofacilitate small-group discussions, promoting medical student participant safety. Students and faculty share program leadership. By having students practice with examples from their lived experiences, our model allows them to develop practical skills and reclaim agency in responding to microaggressions, whether as recipients or witnessing allies. Furthermore, our workshop occurs three times over 24 months, allowing for spaced, repeated practice in recognizing and constructively responding to microaggressions.

## Methods

Our goal was to create a series of workshops to teach communication skills to address implicit bias. The workshop helped participants respond to subtle acts of exclusion that they had experienced, witnessed, or unintentionally initiated, as well as to recognize and decrease their own implicit biases. Participants were evaluated using a retrospective before/after survey at the conclusion of the workshop. The series consisted of two or three interactive workshops over 24 months that addressed constructive responses to microaggressions (Appendix A). If participants attended more than one workshop, we selected their first survey instance to increase comparability across surveys and ensure independence of responses. This resulted in a sample of 180 participants. Ethical approval was granted by the Trustees of Dartmouth College, Committee for the Protection of Human Subjects (Case# STUDY00032299).

### Participants

Study participants were medical and nursing students, residents, fellows, staff, and faculty at the Geisel School of Medicine (Dartmouth), Colby-Sawyer College School of Nursing and Health Professions, Dartmouth Hitchcock Medical Center, and the Veterans Administration Medical Center in White River Junction, Vermont. Student participants from Geisel attended three required workshops while participants from other locations attended invited workshops. Participants were recruited via email (Appendix B) and surveyed (Appendix C).

### Cofacilitators

Two cofacilitators led each small-group workshop. Faculty with experience in facilitation and interest in mitigating implicit bias were invited by student and faculty leaders. However, while prior experience and knowledge were valued, they were not necessary to participate as a cofacilitator. Student cofacilitators were recruited after workshop participation by volunteering or peer recommendation. Cofacilitators were trained in small groups of four to six for 2 hours using a standardized facilitator guide (Appendix D). Cofacilitator trainings were conducted using a hybrid approach (in person and online). Cofacilitator training consisted of sharing facilitation principles for setting up a psychologically brave space for learning, active listening, appreciation, coaching, and the provision of effective feedback. Cofacilitators-in-training facilitated role-play simulations with each other while being coached by faculty. Student and faculty cofacilitators typically participated in more than one training.

### Workshop Timing

Medical students participated in three required 2-hour in-person workshops at the beginning and middle of year 3 and again at the end of year 4. Faculty, staff, residents, and nursing students typically participated in one 2-hour workshop. Participant data were included in analyses if participants attended at least one session and completed at least one survey item.

### Workshop Delivery

The majority of the training used an active learning approach and went beyond a purely cognitive discussion of implicit bias. Each workshop was divided into large- and small-group portions. At the beginning of each workshop, a student (either a participant or a cofacilitator) shared a microaggression they had experienced that exemplified the challenge of responding and the negative impact on the individual. Twenty minutes of speed-meeting activities set the stage for open discussion in small groups. Participants paired up and took turns answering a content-related question about themselves; for example, “Describe an early experience of recognizing difference (in yourself or others).” Participants switched partners for a total of three prompts. The goal was to reflect on how personal narratives reduced implicit bias and to demonstrate participant multidimensionality. Next, a 20-minute didactic provided background information and built community through group learning. The didactic consisted of a PowerPoint presentation (Appendix E) and brief videos ranging in duration from 1 to 3 minutes (Appendices F–H). In subsequent workshops, the didactic portion built on previous knowledge with new content, including stereotype threat, difficult conversations, and effective allyship.

After the didactic, participants received a skills card (Appendix I) and moved to small groups of four to six participants and two cofacilitators. Ground rules were established to acknowledge the sensitivity of discussion topics and set up a brave space. Whereas a safe space would imply that everyone in the room was comfortable, a brave space was an environment where everyone embraced the discomfort that could come with sharing their thoughts, knowing their vulnerability would be met with a nonjudgmental atmosphere. Cofacilitators led introductions where participants shared a part of their identity that brought them strength as well as challenges in the clinical environment. Then, cofacilitators sought a volunteer to set up and debrief role-play simulations for those willing to practice. One person (the responder) assumed the role of the individual receiving the microaggression or the role of bystander/ally. Another participant played the role of the source.^11^ If students were uncomfortable taking on this role, a cofacilitator assumed it. The responder briefly shared the context—the microaggression they had experienced or witnessed—describing relationships and power dynamics. Next, they practiced responding as the receiver or as an ally, choosing one to two skills from the skills card to utilize in their simulation. They also directed how the person playing the source should act and respond. The cofacilitator called a time-out after the scene had played through, usually within 2–3 minutes; sought participant self-reflection; and encouraged participants to practice the same simulation after receiving coaching and feedback, if they felt comfortable doing so. Cofacilitators appreciated strengths in verbal and nonverbal skills and gave one or two points of constructive feedback. If time allowed, cofacilitators solicited other group members for feedback. If a student preferred not to use their own example or had not experienced any microaggressions, they were encouraged to select one from a list of examples experienced by classmates (Appendix J).

At the small group's conclusion, students returned to the large group to share themes or learning points.

### Survey Development

We developed and administered a survey (Appendix C) to evaluate the workshop's impact on participants’ knowledge and comfort in responding to microaggressions. We explored the literature for existing validated measures of these constructs^22^ and developed new survey questions when we did not find appropriate existing measures. Items created de novo distinguished knowledge by role of recipient, source, and ally; community support related specifically to microaggressions; and feelings of safety in speaking up about microaggressions.

Following the workshop, we sent participants via email a 10-minute online Qualtrics survey consisting of preworkshop and postworkshop questions. We collected surveys between September 2022 and June 2023. Our survey featured 66 total items, which included 22 items repeated as both preworkshop and postworkshop questions as well as some questions for students only or faculty/staff only. There were 10 demographic items; four items asking about participants’ prior experience with microaggressions (directed toward themselves, a student, a staff/faculty member, or a patient); five items about challenges participants faced in addressing microaggressions, three of which were de novo questions; four items assessing comfort in addressing microaggressions, all de novo; six items assessing knowledge/skills in addressing microaggressions, three de novo; one item asking about participants’ prior knowledge of microaggressions; items assessing opportunities to discuss microaggressions in the community (one), de novo, and safety in speaking up about microaggressions in different settings (three for students, four for staff/faculty), four de novo; and three items identifying how many people participants felt they could speak to about microaggressions, all de novo.

The items assessing comfort, knowledge/skills, and people participants could speak to were asked once as a postworkshop question and once as a retrospective preworkshop question.^23^ This type of question allowed for all items (retrospective pretest or “before” items and posttest or “after” items) to be included on one survey instance and addressed potential response shift bias.^24^ Prior knowledge was assessed using a single-item, 4-point scale.

### Statistical Analysis

The data were analyzed using IBM SPSS Statistics for Mac Version 28.0. Separate exploratory factor analyses using generalized least squares extraction and promax rotation yielded a one-factor solution for comfort (four items) and a one-factor solution for knowledge (six items). Reliability of the resulting scales was adequate (comfort: α = .68–.87; knowledge/skills: α = .84–.93). As a result, we created an average scale score for comfort (range: 1–4) and an average scale score for knowledge/skills (range: 1–4) incorporating all the respective items.

Descriptive statistics, means, and frequencies were used to explore participant survey data. Two separate factorial analyses of variance (ANOVAs) compared the main effects of time (before/after), role (student vs. staff/faculty), and prior knowledge. The interaction effect between time and role and the interaction effect between time and prior knowledge on the comfort and knowledge average scale scores were conducted to assess the impact of the workshops. A power analysis indicated there was power to detect a medium effect for all analyses. All assumptions for analyses were met, unless otherwise indicated.

## Results

Survey participants’ average age was 34.7 years, the average age of students was 27.3 years, and the average age of faculty/staff was 47.4 years (Table 1). Students and faculty/staff comprised 62% (*n* = 112) and 38% (*n* = 68) of participants, respectively. About half (*n* = 91) identified as women, 35% (*n* = 63) as men, and 2% (*n* = 3) as nonbinary. Participants were predominantly White (70%, *n* = 126), and most (89%, *n* = 160) had received college-level education or higher. Participants who completed the workshop reported greater knowledge (before *M* = 2.5, after *M* = 2.8, *p* < .001) and comfort (before *M* = 2.3, after *M* = 2.5, *p* < .001) identifying and responding to microaggressions (Table 2).

**Table 1.**
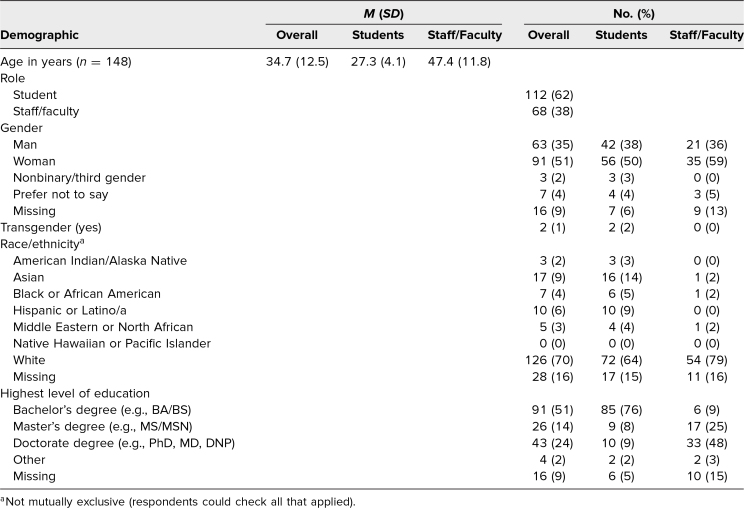
Workshop Survey Demographic Information

**Table 2.**
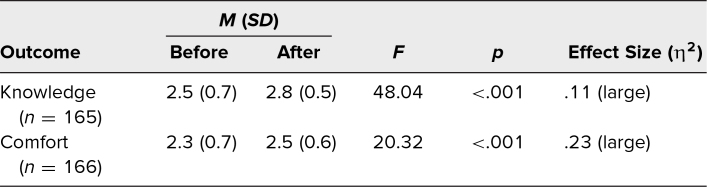
Participants’ Overall Means Before and After the Workshop

For the factorial ANOVA with knowledge as the dependent variable, the main effects of time (*p* < .001) and prior knowledge (*p* < .001) were significant and displayed large effects. The interaction effects between time and role (*p* < .01) and between time and prior knowledge (*p* < .01) were significant and displayed small to medium effects (small effect for time and role, medium effect for time and prior knowledge). For the factorial ANOVA with comfort as the dependent variable, the main effects of time (*p* < .001) and prior knowledge (*p* < .001) were significant and displayed large effects. The interaction effects between time and role (*p* < .05) and time and prior knowledge (*p* < .05) were significant and displayed small to medium effects (small effect for time and role, medium effect for time and prior knowledge). These results are summarized in Table 3.

**Table 3.**
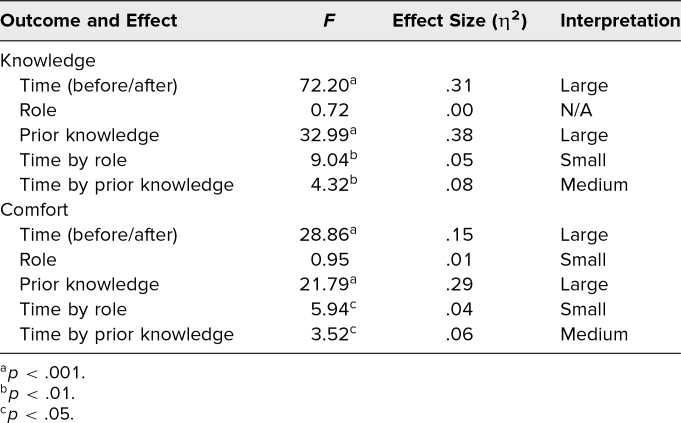
Workshop Survey Results of Factorial Analyses of Variance With Knowledge and Comfort as Outcomes

Since the main effect of time was significant for the outcomes of knowledge and comfort, this suggests that knowledge of and comfort with microaggressions both increased postworkshop as compared to preworkshop. Since the main effect of previous knowledge was significant for these same outcomes, this suggests that those with the least previous knowledge of microaggressions had the most to gain from the workshop. Finally, the interaction effects support these conclusions as time (pre- vs. postworkshop) and its effects on knowledge and comfort were clearly related to how much prior knowledge a participant had (medium effect).

Participants were also surveyed about people available to support them with experiences of microaggressions. A higher number of participants reported having more people to support them postworkshop compared to preworkshop (Table 4). Notably, a few participants reported having fewer people to ask for support postworkshop compared to preworkshop (Table 4).

**Table 4.**
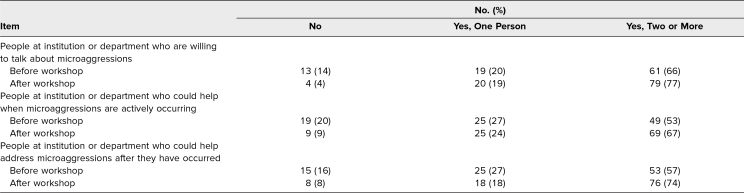
Percentage of Participants Before and After the Workshop Who Could Identify People Available to Support Them

## Discussion

As a result of our workshop, participants reported greater knowledge, skills, and comfort in responding to microaggressions and increased ability to speak up, when safe to do so. The data demonstrate that participants with less exposure to implicit bias education benefited most from this training. This underscores the importance of training all members of a medical community to ensure that those who would most benefit from the training are involved and that everyone, regardless of prior knowledge of implicit bias, leaves the workshop with a shared vocabulary and understanding. Previous research has described using a senior medical student and a staff/faculty member co-teach^19^ or a senior resident and staff/faculty member co-teach.^18^ Our work is unique as students and staff/faculty cocreated the curriculum and cofacilitated the workshops, providing a tailored perspective of student and staff/faculty experience.

The standardized facilitator guide used in training cofacilitators is unique to our knowledge. In this workshop, the majority of time focuses on active small-group learning via skills practice rather than large-group didactics, as research has indicated that more active learning in small groups creates lasting change.^14^ Including the student voice in program design allowed greater relevance for student participants and increased staff/faculty awareness of microaggressions that students were most likely to encounter. We present a model that other health professional training programs and academic medical centers can replicate and tailor to their specific communities. While we acknowledge that brief interventions may not lead to sustained outcomes, we aim to mitigate this risk by holding multiple phased workshops over 24 months to reinforce skills over time. Including a wide range of real-life scenarios (where sources include staff/faculty and patients) may expand the applicability of skills practiced across diverse health professions academic settings. Moreover, witnessing peers/colleagues respond to microaggressions in a simulation encourages perspective taking. Lastly, we train groups of participants within their medical community, such as training the emergency department or all student coaches together, in order to create a culture of change and ensure group members develop shared vocabulary around communication skills.

While our results are robust, some limitations exist. First, while retrospective pre/post methodology reduces response shift bias, it can potentially introduce other biases, such as social desirability bias or recall bias. While the exposure to training presented opportunities for participants and cofacilitators to hone their knowledge and skills and reflect on their perceptions and reactions to addressing microaggressions, no causal mechanism was established. Our sample size was large enough to detect a medium effect in our data, and we saw a large effect on increased comfort and knowledge postworkshop. However, only small to medium effects for the interaction between time and role and the interaction between time and prior knowledge were detected. Additionally, the majority of participants were White and located in a rural community, so results may not be generalizable to all US academic medical centers.

While this workshop has many benefits, we recognize that there exists a potential for harm. A few participants reported feeling they had more people to ask for help with microaggressions prior to participating in a workshop compared to afterward. This could be an inaccurate artifact due to the nature of self-reported data or to potentially triggering experiences that may have occurred if role-playing was done using personal experiences. Reliving traumatizing experiences in front of peers who may react differently or who do not yet have skills to respond in an empathetic manner can potentially harm participants. This can be augmented by the power dynamic of a staff/faculty member giving a student participant feedback when their lived experience is different.

Given these concerns, we prioritized cultural humility, background in implicit bias, and gentle communication skills in choosing and training staff/faculty and student cofacilitators to minimize risk of harm. We clearly stated to both facilitators and participants that participants’ level of engagement was entirely their decision. Additionally, we acknowledged that each participant's past experiences in life and throughout the course of the day leading up to the workshop might influence how they received the workshop. Facilitators were trained to lead participants through exercises to uncover solutions that would be meaningful to and work for the participant rather than advocating for any particular outcome or method during skills practice. We also recognized that, while we aimed to create a brave space in small groups, participants had relationships with one another that existed outside of this context, which could affect their experiences before, during, and after the workshop.

While research has examined the self-reported growth of student workshop participants, few data compare staff/faculty and student participant outcomes or examine workshop facilitator outcomes. We are currently examining data on the cofacilitator experience and comparing staff/faculty and student workshop results. A future workshop protocol could start training medical students as early as their first year. Studies have found that early intervention may contribute to earlier culture change.^3^ Finally, a follow-up assessment could measure whether participants use these skills in practice in the learning environment and if the workshop has sustained impact on comfort with and knowledge of microaggressions over time.

## Appendices


Description of Microaggressions Workshop.docxEmail Advertisement.docxSurvey.docxCofacilitator Guide.docxLarge-Group Presentation.pptxGender Bias Simulation.mp4Student in Wheelchair Simulation.mp4Nursing Student Simulation.mp4Skills Card.docxMicroaggression Examples.docx
*All appendices are peer reviewed as integral parts of the Original Publication.*
