# Supplementary material for: What to Say When It Matters: Communication Skills to Address Implicit Bias Workshop
Source: MedEdPORTAL. 2025 Apr 15;21:11514. doi: 10.15766/mep_2374-8265.11514 (PMC11997152; doi:10.15766/mep_2374-8265.11514)
Supplement: Supplementary file 1 — Description of Microaggressions Workshop.docxEmail Advertisement.docxSurvey.docxCofacilitator Guide.docxLarge-Group Presentation.pptxGender Bias Simulation.mp4Student in Wheelchair Simulation.mp4Nursing Student Simulation.mp4Skills Card.docxMicroaggression Examples.docx [file mep_2374-8265.11514-s001.zip › A. Description of Microaggressions Workshop.docx]

**Communication Skills to Address Implicit Bias**

**Overview:** The goal of the workshops is to empower students, faculty, and staff in clinical and learning environments, to recognize and constructively respond when they experience, witness, or unintentionally engage in subtle acts of exclusion, as well as to recognize and decrease their own implicit biases.

Subtle acts of exclusion, or microaggressions, are experienced commonly by BIPOC, female, LGBTQ+, and people living with (visible and invisible) disabilities, among others, in our clinical and learning environments. They go beyond verbal abuse and may include general disrespect, invalidation, and even the exclusion of targeted individuals (e.g. assigning a team member responsibilities commonly associated with gender stereotypes, assuming someone’s ability based on race or accent).

In the past, subtle acts of exclusion have often been ignored and their adverse impact minimized. This can increase the impact of stereotype threat, which is known to decrease a sense of belonging, negatively impact performance, and increase burnout. Failure to recognize and respond effectively to subtle acts of exclusion is often due to lack of awareness, lack of self-confidence to speak up, lack of role modeling, and inadequate communication skills to engage in difficult conversations in a productive manner that maintains relationships with complex power dynamics.

**Workshop Details:** Students typically participate in two 2-hour workshops. The bulk of the training uses an active learning approach - participants are introduced to and then practice skills to engage constructively in these difficult conversations.

After community building and a short didactic in a large group, participants receive a skills card and practice role plays in a small group of 4-6 participants with trained facilitators (usually a faculty and a student co-facilitate). The simulations may be based on a microaggression experienced by a participant or can be selected from a list of real examples from the same community as the participants. Coaching and feedback are provided in small, psychologically brave groups and specific skills to approach these situations are discussed. Having 2 workshops spaced over 2-3 months provides time for participants to practice skills and reflect on the challenges between sessions, and to return to learn more and practice again.

Participants who have finished the training report greater self-confidence and empowerment in responding to subtle acts of exclusion and increased ability to speak up, when it is safe to do so.
