# Supplementary material for: What to Say When It Matters: Communication Skills to Address Implicit Bias Workshop
Source: MedEdPORTAL. 2025 Apr 15;21:11514. doi: 10.15766/mep_2374-8265.11514 (PMC11997152; doi:10.15766/mep_2374-8265.11514)
Supplement: Supplementary file 1 — Description of Microaggressions Workshop.docxEmail Advertisement.docxSurvey.docxCofacilitator Guide.docxLarge-Group Presentation.pptxGender Bias Simulation.mp4Student in Wheelchair Simulation.mp4Nursing Student Simulation.mp4Skills Card.docxMicroaggression Examples.docx [file mep_2374-8265.11514-s001.zip › B. Email Advertisement.docx]

Dear ______,

The **Communication Skills to Address Implicit Bias team** hosts workshops for medical students and other hospital team members to learn skills to respond to microaggressions in medical settings and create a more supportive, welcoming community free of bias. We host 90-minute workshops throughout the year, involving small groups led by facilitators to discuss scenarios, identify skills, and role-play approaches to difficult conversations.

**We are seeking new student co-facilitators. Your role is key since students experience psychological safety when a fellow student is in that role.**

Students co-facilitate with a trained faculty or staff member. The workshops consist of a student story, a speed meeting, and a brief didactic followed by 90 minutes of group practice.

At the end of the session, students should be able to:

1. Name and practice responses to subtle acts of exclusion
2. Begin to notice how power and status impact team functioning
3. Define stereotype threat
4. Discuss strategies to mitigate stereotype threat

**The workshops will take place on the following dates/times:**

**___________**

**___________**

**Please let us know if you have any questions! Sign up to co-facilitate here (insert your own Google doc/survey link).**

Thank you,

Communication Skills to Address Implicit Bias Team

Note: the above sample recruitment email was drafted based on similar emails from Dartmouth Communication Skills to Address Implicit Bias Team and Amanda Philpot BS, MSc.
