# Supplementary material for: What to Say When It Matters: Communication Skills to Address Implicit Bias Workshop
Source: MedEdPORTAL. 2025 Apr 15;21:11514. doi: 10.15766/mep_2374-8265.11514 (PMC11997152; doi:10.15766/mep_2374-8265.11514)
Supplement: Supplementary file 1 — Description of Microaggressions Workshop.docxEmail Advertisement.docxSurvey.docxCofacilitator Guide.docxLarge-Group Presentation.pptxGender Bias Simulation.mp4Student in Wheelchair Simulation.mp4Nursing Student Simulation.mp4Skills Card.docxMicroaggression Examples.docx [file mep_2374-8265.11514-s001.zip › C. Survey.docx]

# Responding to Microaggressions: Workshop Survey

Thank you for participating in this workshop and for filling out this survey! We will use the information you provide to assess and improve our workshops. Your survey is confidential, meaning no information you provide will be linked back to you, and any summaries of data from this survey will be presented in aggregate with all other respondents’ data.

1. [Display if role is not predetermined] What is your role at [Insert Institution] or your clinic or work setting?
   - Medical student
   - Resident or fellow
   - Staff or faculty
2. What was the date of the workshop you participated in?
   - [Workshop dates]
3. Thinking about your time at [Insert Institution] or your clinical department, have you experienced or witnessed microaggressions directed towards:

|  | No, never | Yes, once | Yes, more than once |
| --- | --- | --- | --- |
| Yourself | ⚪ | ⚪ | ⚪ |
| A student | ⚪ | ⚪ | ⚪ |
| A staff/faculty member | ⚪ | ⚪ | ⚪ |
| A patient | ⚪ | ⚪ | ⚪ |

1. How challenging are each of the following for you in addressing microaggressions?

|  | Not at all challenging | Somewhat challenging | Moderately challenging | Very challenging |
| --- | --- | --- | --- | --- |
| Limited knowledge of how to address the comment/behavior | ⚪ | ⚪ | ⚪ | ⚪ |
| Lack of visible allies present who will support you if you speak up | ⚪ | ⚪ | ⚪ | ⚪ |
| Lack of familiarity with support systems at [Insert Institution] or clinical department to address issue | ⚪ | ⚪ | ⚪ | ⚪ |
| Discomfort speaking with the source of the comment/ behavior | ⚪ | ⚪ | ⚪ | ⚪ |
| *[Display to: faculty and staff]* Lack of knowledge on how to be an effective ally | ⚪ | ⚪ | ⚪ | ⚪ |

1. How comfortable would you be in each of these scenarios?

|  | Not at all comfortable | Somewhat comfortable | Moderately comfortable | Very comfortable |
| --- | --- | --- | --- | --- |
| Talking about microaggressions at [Insert Institution] or clinical department | ⚪ | ⚪ | ⚪ | ⚪ |
| *[Display to: students/residents/fellows]* Responding as an ally when a microaggression is occurring | ⚪ | ⚪ | ⚪ | ⚪ |
| *[Display to: faculty and staff]* Responding as an ally when a microaggression is occurring against a student or team member | ⚪ | ⚪ | ⚪ | ⚪ |
| Responding as a recipient when a microaggression is occurring | ⚪ | ⚪ | ⚪ | ⚪ |
| Responding as the source when you have engaged in a microaggression | ⚪ | ⚪ | ⚪ | ⚪ |
| *[Display to: faculty and staff]* Responding to a student or team member who tells you they experienced a microaggression | ⚪ | ⚪ | ⚪ | ⚪ |

1. How would you rate yourself on the following?

|  | Poor | Fair | Good | Excellent |
| --- | --- | --- | --- | --- |
| Ability to define the term “microaggression” | ⚪ | ⚪ | ⚪ | ⚪ |
| Ability to notice when a microaggression is occurring | ⚪ | ⚪ | ⚪ | ⚪ |
| *[Display to: students/residents/fellows]* Knowledge of how to respond as an ally when a microaggression is occurring |  |  |  |  |
| *[Display to: faculty and staff]* Knowledge of how to respond as an ally when a microaggression is occurring against a student or team member | ⚪ | ⚪ | ⚪ | ⚪ |
| Knowledge of how to respond as a recipient when a microaggression is occurring | ⚪ | ⚪ | ⚪ | ⚪ |
| Knowledge of how to respond as the source when you have engaged in a microaggression | ⚪ | ⚪ | ⚪ | ⚪ |
| *[Display to: faculty and staff]* Knowledge of how to respond to a student or team member who tells you they experienced a microaggression | ⚪ | ⚪ | ⚪ | ⚪ |
| Overall preparedness to address microaggressions | ⚪ | ⚪ | ⚪ | ⚪ |

1. Are there people at [Insert Institution] or your clinical department who…

|  | No | Yes, 1 person | Yes, 2 or more people |
| --- | --- | --- | --- |
| …are willing to talk to you about microaggressions | ⚪ | ⚪ | ⚪ |
| …can help you when microaggressions are actively occurring | ⚪ | ⚪ | ⚪ |
| …can help you address microaggressions after they have occurred | ⚪ | ⚪ | ⚪ |

1. [Insert Institution] leaders are interested in knowing about how they can best meet the needs of students, faculty and staff. This workshop represents one opportunity to discuss microaggressions. How would you rate the availability of opportunities to discuss microaggressions in the [Insert Institution] or clinical department communities?
   - Poor, there are **no** other opportunities
   - Fair, there are **1 or 2** other opportunities
   - Good, there are **a few** other opportunities
   - Excellent, there are **many** other opportunities
2. How often do you feel safe to speak up when microaggressions occur in the following settings?

|  | Never | Rarely | Sometimes | Often | Almost always | Not applicable |
| --- | --- | --- | --- | --- | --- | --- |
| Clinical settings | ⚪ | ⚪ | ⚪ | ⚪ | ⚪ | ⚪ |
| Educational settings | ⚪ | ⚪ | ⚪ | ⚪ | ⚪ | ⚪ |
| *[Display to: faculty and staff]* Working environment (non-clinical/non-educational environment) | ⚪ | ⚪ | ⚪ | ⚪ | ⚪ | ⚪ |
| Personal life | ⚪ | ⚪ | ⚪ | ⚪ | ⚪ | ⚪ |

1. What is a strategy you plan to use to address microaggressions in the future?

____________________________________________________

1. How likely are you to attend future trainings or workshops on how to handle microaggressions?
   - Very unlikely
   - Unlikely
   - Likely
   - Very likely
2. What additional training opportunities would you find helpful?

__________________________________________________________________

Now we’d like you to do something a little different. Think about how you felt and what you did *before* this workshop, a week ago. Please answer the following questions about how you felt and what you did *before* this workshop.

1. Before you participated in this workshop, how familiar were you with the term microaggression?
   - I had never heard it
   - I had heard it, but could not define it
   - I could define it, but could not identify when it was happening
   - I could define it and identify it when it was happening
   - I could define it, identify when it was happening, and knew how to intervene
2. Before you participated in this workshop, how challenging were each of the following for you in addressing microaggressions?

|  | Not at all challenging | Somewhat challenging | Moderately challenging | Very challenging |
| --- | --- | --- | --- | --- |
| Limited knowledge of how to address the comment/behavior | ⚪ | ⚪ | ⚪ | ⚪ |
| Lack of visible allies present who would support you if you spoke up | ⚪ | ⚪ | ⚪ | ⚪ |
| Lack of familiarity with support systems at [Insert Institution] or clinical department to address issue | ⚪ | ⚪ | ⚪ | ⚪ |
| Discomfort speaking with the source of the comment/behavior | ⚪ | ⚪ | ⚪ | ⚪ |
| *[Display to: faculty and staff]* Lack of knowledge on how to be an effective ally | ⚪ | ⚪ | ⚪ | ⚪ |

1. Before this workshop, how comfortable were you in each of these scenarios?

|  | Not at all comfortable | Somewhat comfortable | Moderately comfortable | Very comfortable |
| --- | --- | --- | --- | --- |
| Talking about microaggressions at [Insert Institution] or clinical department | ⚪ | ⚪ | ⚪ | ⚪ |
| Responding as an ally when a microaggression was occurring against a student or team member | ⚪ | ⚪ | ⚪ | ⚪ |
| Responding as a recipient when a microaggression occurred | ⚪ | ⚪ | ⚪ | ⚪ |
| Responding as the source when you had engaged in a microaggression | ⚪ | ⚪ | ⚪ | ⚪ |
| *[Display to: faculty and staff]* Responding to a student or team member who told you they experienced a microaggression | ⚪ | ⚪ | ⚪ | ⚪ |

1. How do you rate yourself, before the workshop, on the following?

|  | Poor | Fair | Good | Excellent |
| --- | --- | --- | --- | --- |
| Ability to define the term “microaggression” | ⚪ | ⚪ | ⚪ | ⚪ |
| Ability to notice when a microaggression occurred | ⚪ | ⚪ | ⚪ | ⚪ |
| Knowledge of how to respond as an ally when a microaggression was occurring against a student or team member | ⚪ | ⚪ | ⚪ | ⚪ |
| Knowledge of how to respond as a recipient when a microaggression occurred | ⚪ | ⚪ | ⚪ | ⚪ |
| Knowledge of how to respond as the source when you had engaged in a microaggression | ⚪ | ⚪ | ⚪ | ⚪ |
| *[Display to: faculty and staff]* Knowledge of how to respond to a student or team member who told you they experienced a microaggression | ⚪ | ⚪ | ⚪ | ⚪ |
| Overall preparedness to address microaggressions | ⚪ | ⚪ | ⚪ | ⚪ |

1. Before the workshop, would you have said that there were people at [Insert Institution] or your clinical department who…

|  | No | Yes, 1 person | Yes, 2 or more people |
| --- | --- | --- | --- |
| …were willing to talk to you about microaggressions | ⚪ | ⚪ | ⚪ |
| …could help you when microaggressions were actively occurring | ⚪ | ⚪ | ⚪ |
| …could help you address microaggressions after they had occurred | ⚪ | ⚪ | ⚪ |

Thank you for your answers so far. You’re almost done! We just have a few more questions about you so that we can describe who was in our workshop. Remember that the information you provide in this survey is confidential and your responses will not be linked to you individually.

1. What is your age? Please enter a whole number in years. _____ (*Validation for whole number*)
2. What is your gender?
   - Male
   - Female
   - Non-binary/third gender
   - I use another term (____)
   - Prefer not to say
3. Do you identify as transgender?
   - Yes
   - No
   - Prefer not to say
4. Which of the following best describes your identity? Select all that apply.
   - American Indian or Alaska Native
   - Asian
   - Black or African American
   - Hispanic or Latino/a
   - Middle Eastern or North African
   - Native Hawaiian or Pacific Islander
   - White
5. What languages are you fluent in, aside from English? ______
6. *[Display to: faculty and staff]* What is your role at [Insert Institution] or your clinical department (check all that apply)?
   - Direct clinical care
   - Teaching
   - Research
   - Administration
   - Other (please specify: ___)
7. *[Display to: students]* What graduate level training program are you currently enrolled in, if any?
   - MD/DO
   - Nursing
   - APRN
   - PA
   - MPH
   - Other (please specify: ___)
8. What is your highest level of education that you have completed?
   - Bachelor’s degree (e.g., BA/BS)
   - Master’s degree (e.g., MS/MSN)
   - Doctorate degree (e.g., PhD/MD/DNP)
   - Other (please specify: ___)
9. Do you have any other thoughts or suggestions about the workshop that you have not provided above?

___________________________________________________

***[If distributed with an anonymous link]*** Please fill out the following (optional) information so that we know who has already completed the survey and who may need follow-up.

First name: __________

Last name: ________

Email address: ________

Thank you for completing this survey! You are finished.

**CME questions**

1. How effective was this learning activity at meeting the following learning outcome?

|  | Not at all effective | Slightly | Moderately | Very effective |
| --- | --- | --- | --- | --- |
| Participants will be able to describe at least three important factors that must be considered when coaching students from underrepresented populations with identities or backgrounds that are different from their own. | ⚪ | ⚪ | ⚪ | ⚪ |

1. To what extent did this learning activity meet the following criteria?

|  | Not at all | Slightly | Moderately | Very | N/A |
| --- | --- | --- | --- | --- | --- |
| The activity was grounded in science and/or was evidence-based | ⚪ | ⚪ | ⚪ | ⚪ | ⚪ |
| The activity was free from commercial bias | ⚪ | ⚪ | ⚪ | ⚪ | ⚪ |

1. As a result of your participation in this learning activity, how will you integrate a change(s) into your practice or behavior?

__________________________________________________

1. How well did this learning activity meet the following aspects of interprofessional continuing education (IPCE)?

|  | Not at all well | Slightly | Moderately | Very well | N/A |
| --- | --- | --- | --- | --- | --- |
| Demonstrating mutual respect for the other healthcare professionals | ⚪ | ⚪ | ⚪ | ⚪ | ⚪ |
| Defining the roles and responsibilities of my team members | ⚪ | ⚪ | ⚪ | ⚪ | ⚪ |
| Engaging in effective interprofessional communication | ⚪ | ⚪ | ⚪ | ⚪ | ⚪ |
| Working within an interprofessional team | ⚪ | ⚪ | ⚪ | ⚪ | ⚪ |

1. Explain how you will share the information provided during this learning activity with your interprofessional team in order to develop a plan to improve patient care.

__________________________________________________
