# Supplementary material for: What to Say When It Matters: Communication Skills to Address Implicit Bias Workshop
Source: MedEdPORTAL. 2025 Apr 15;21:11514. doi: 10.15766/mep_2374-8265.11514 (PMC11997152; doi:10.15766/mep_2374-8265.11514)
Supplement: Supplementary file 1 — Description of Microaggressions Workshop.docxEmail Advertisement.docxSurvey.docxCofacilitator Guide.docxLarge-Group Presentation.pptxGender Bias Simulation.mp4Student in Wheelchair Simulation.mp4Nursing Student Simulation.mp4Skills Card.docxMicroaggression Examples.docx [file mep_2374-8265.11514-s001.zip › D. Cofacilitator Guide.docx]

**What to Say When it Matters: Communication Skills to Address Implicit Bias**

Co-Facilitator Guide

**Background:** Developing diverse, equitable, welcoming medical learning environments where every student feels they belong and are supported is essential to enhance learning.

**Goals:** Engage medical students in an interactive workshop that emphasizes equity and inclusion and provides communication skills training that facilitates effective dialogue about differences. Students will be able to recognize subtle acts of exclusion (microaggressions) and practice responding constructively using new skills in a safe learning environment.

**Brief Description**: Students will engage in a 2 hour workshop that includes team building exercises, and communication skills training for equity and allyship, with practice time in small groups. All levels of experience and knowledge in diversity, equity and inclusion are welcome.

**Detailed Agenda**

| **SESSION** | **Min** | **OVERVIEW** |
| --- | --- | --- |
| Main Session 1:  50” | 10” | **WELCOME and Introduction:**  Leaders introduce themselves  Student shares a story about implicit bias. Or read an example (Appendix J)  **Goal: work towards learning environments that respect and welcome all forms of diversity** |
|  | 20” | **Speed-meeting** in groups of 2, speaking for 90 sec” each  If you are the listener: listen silently and attentively, only ask clarifying questions.  Each person in a pair answers the same question, then you switch to a new partner, ideally in your small group  **Debrief -** **Goal: experience and reflect on how personal narratives reduce implicit bias. “Teamwork before task work”** |
|  | 20” | **Didactic:** Leader Presents Powerpoint (Appendix E) and Video (Appendices F-H)  Workshop MS2: Focus on difficult conversations, implicit bias, allyship skills, review microaggressions card and small group process  Workshop MS3: Focus on Ladder of Inference, stereotype threat, allyship skills  Use a brief video from appendices F-H as an example of addressing a microaggression to stimulate discussion on what was done well and what could be improved.  **Goal: Introduce evidence-based skills that demonstrate respect and earn trust across difference** |
| Small Groups | 95” | **Small Group**: (includes 5” bio break before starting in a small group room).  You will have a faculty co-facilitator  You will work with a small group of 4-6 student participants  **Co-facilitating with your co-facilitator will require you to:**  **1. Prepare together in advance**  2. Set up a psychologically safe group to practice.  3. Manage time efficiently so all get a chance to practice, and not ***just talk*** about microaggressions. The goal is for all to learn and practice new skills.  4.Ensure that each microaggression chosen for practice is clear and focused enough for useful skills practice.  5. Ensure that the student in the “hot seat” chooses 1-2 communication skills to practice before role play starts (from the card we provide)  6.Pay attentive to subtle nonverbal communication in addition to language – if someone does not want to participate, they can engage as an observer. If they are triggered, explain counseling options (talk with Nan, Alison, their coach or Dick’s House counseling)  7. Call for a ‘time’ out after the communication skill chosen has been practiced. Debrief the ‘hot seat’ participant first “how did that feel?” “what did you do well?” then ask “what do you want to do differently next time?”  7. Provide lots of positive reinforcement for skills they do well.  8. Give constructive feedback clearly explaining what they might try to do better. Ask them to do it a second time! |
| Main Session 2: | **15”** | **Closing:**  Harvest Learnings  Commit to practicing a new skill |
|  |  |  |

**Co-Facilitator Guide for Small Groups**

**Welcome** (1 minute)*: “Welcome to our ~ 90 minute small group - thank you for being here! This group is meant to engage us in conversations that build community and to invite you to practice skills that are useful in responding to subtle acts of exclusion. My co-facilitator and I will provide coaching, appreciation and feedback.”*

**Group Agreements** (5 minutes): This is very sensitive work we will be doing together that often makes people feel vulnerable.

“*Can we agree that while you are encouraged to share what you learn from the group, that the stories other people share will be kept private and confidential?”*

*“Are there other group agreements you want to suggest?” These below were covered in the large group.*

- *Speak for yourself*
- *Assume good intent*
- *Openness to learning from each other*
- *No fixing*
- *Name any subtle act of exclusions that occur (often called oops/ouch rule)*
- *When the going gets rough, turn to wonder (rather than ignore it)*

**Ask for a timekeeper to divide the time equally and a reporter who will report back in large group 2-3 of the takeaways from small group.**

**Ask for a recorder to record themes to report back to the large group, not specific examples.**

**Introductions of group members**: (20 minutes) to share aspects of their identity and privilege.

*“I invite all of us to introduce yourselves. You will have 2 minutes to introduce yourself – please speak to one other person who you will name before you start. I will keep track of time. Please tell the listener something about one of your identities (that you are comfortable sharing) that is a strength. This may be associated with privilege, or with challenge or with both. Also, feel free to share aspects which have informed your work in the DEI sphere. The listener will then become the speaker and will introduce themselves to another group member (who they identify before starting to speak), until everyone has spoken. The last student will introduce themselves to one of the co- facilitators.*

***The way that you as co- facilitator introduce yourself at the beginning sets the stage for others*** *so please discuss an important identity, including mentioning any privilege you have.*

**Introduction to skills card:** Hand out the skills card (Appendix I) and explain that it features a variety of methods that may be used to address microaggressions both as a recipient and as an ally. Different skills may be more appropriate for different situations. Different skills may also feel more natural for one person as compared to another.

**Introduction to skills practice** *(3”): “I wish that bias did not happen here or anywhere. We know it does and we will be working on responding to expressions of bias that you have witnessed or experienced. No one should have to be alone with these experiences* *and* m*aking the invisible visible can lighten the load. The goal is* ***not to re-trigger you*** *so please only share what you are comfortable sharing.”*

*“Let’s make the most of the time we have as we practice skills that can help us all become better at speaking up and allying with fellow students and patients. Awareness and skills are crucial to being an ally. Practicing these skills is really important and the time we have today to actually practice skills is rare. I’d like someone who is feeling brave to volunteer to go first.”*

*If someone declines your invitation to practice a microaggression, do not pressure them. Instead, say something like, “It is fine to pass, we welcome your active engagement and will ask you to share your observations*.” I recommend circling back to them, giving them another chance later since they often change their mind once they see others’ simulations.

**Set up Skills Practice** (~ 5 minutes – this takes longer for the first role play than subsequent ones):

**Goal: Practice communication skills to constructively respond to a microaggression using 1-2 skills from the card (which you will review together).**

*“I’m inviting all of you to choose a simulation to practice. You may want to use one that you have personally experienced or witnessed that you wish you had responded to differently. Or if you prefer, you can choose one from the list of microaggressions experienced by other students. The goal is to help you reclaim your voice and be better prepared the next time one occurs. We don’t expect you to actually speak up where it would not be safe to respond, for example one in which the source of the microaggression is a superior who will be evaluating you. However, it takes a lot of practice over years to get comfortable speaking up and this is a chance to practice in a safe(r) space.*

*Please refer to your skills card and choose 1-2 skills to use in your role play. As you can see, there are skills to practice responding directly to microaggressions listed on one side, and allyship skills listed on the other side.*

**In your setup, be as efficient as possible. Ask the student to BRIEFLY share the “back-story”, i.e. clarify context, prior relationship with the person in the opposing role, hierarchy/power issues, how they want the “other role” played and allow them to choose someone to play the opposing role in their scenario. I suggest giving the “source” a different name than their own.**

- **Emphasize the “*play*” in role play – the goal is NOT to be perfect but to try new things, and try again!**
- **Ask if they want to practice responding directly to a microaggression or to respond as an ally**
- **Ask the student to choose 1-2 skills from the skills card to practice in their simulation - have them use the card during the simulation if they want sample language.**
- **Ask the student to decide how intense they want the other character in the role play to act.** On a scale from 1 to 10 we usually recommend starting with a 5/10 level of difficulty.

**Remind the student in the hot seat that the** **focus is to *practice new skills*.**

**- Time simulation for 3” but allow it to extend if rich learning is happening.**

- **Ask observers** in each simulation to take notes so they can give specific, behaviorally based feedback.

**- When to call a timeout?** Sooner than you think!

- if the learner is struggling or gets stuck – no one wants to look deskilled.
- soon after the communication skill planned in advance has been practiced.
- if there is a pertinent teaching point you’d like to make that can’t wait
- if the student has accomplished their goal
- at the prescribed time limit
- if the group or any student begins to laugh - ask “why are *we* laughing?”. Make sure no one feels humiliated.

In the time out, ask how the student is doing, and if time, elicit brief suggestions from observers about how to move forward. Then allow the student to continue, if they choose. You can also ask if another student wants to step in and make it a “rolling” role play if the original student wants to stop and you feel there is more learning to explore.

**When Debriefing (both in time outs and at end if you have time)**:

1. **Check in with the person** in the hot seat first (sometimes this is the only person you check in with)
   - How’s it going? How are you doing/feeling? (emotional check-in)
   - What did you do well? (try to prevent the inevitable drift into self-criticism)
   - What would you like to try to do differently? Encourage them to try again if time allows.
2. **Check in briefly with the person in the other role** next.

- What did the student do well?
- What was your emotional reaction to what they said? (and If relevant, to the NV messages)
- What might they try to do differently?

1. **Check in with observers - only IF YOU HAVE TIME:** Observers often have a great deal of feedback, and it is frequently necessary to limit the amount of feedback to one or two points per observer. Don’t call on people if you feel that most of the feedback and learning has already organically come up.
2. **Save your feedback for last**. Pay attention to how much feedback the interviewer has received and prioritize your most important feedback. It may be useful to ask the interviewer how much feedback they can hear at the moment, and give them the opportunity to limit the feedback.
3. **Offer to rewind and replay the exercise -** if necessary tighten up the role play to enable the time to be spent on practicing the skill(s) at hand. This is a very important thing to remember**; the more re-playing, with an explicit focus on the skills being practiced**, the better.
4. **Consider asking another participant to be in the hot seat** (the **“rolling”** role play), or switch roles for each person practicing so they can feel what it’s like to be on the other side of the coin. You may also choose to switch scenarios.

**Debrief at end of simulations:** If the following issues haven’t come up organically in your discussion, try to discuss them before the end of your small group if there is time.

1. Should I respond now/later/not at all? Should it be done in private or in a subgroup or in the full group, or should I discuss with the clerkship coordinator, or another authority figure. Emphasize that we know they wouldn’t speak up to an attending at this stage of training, however, it is useful to practice speaking up skills since they might speak up with other team members or patients.
2. Who should I ally with? Who else? How is this best done without stepping on their toes?
3. Effect of power and hierarchy – what are the repercussions of speaking up? Fear of retaliation, receiving a poor evaluation, marginalization.
4. Avoid/minimize shaming or humiliating the source of the microaggression
5. Levity vs gravity in one’s response

**Closing** (10 minutes) Discuss any insights and learnings before returning to the large group – the student who has kept time will tell you when to return to the large group!

*“There is a lot more to share - this is just a beginning. These skills require lifelong practice! To close, I’m asking for each one of you to summarize in one phrase what you are leaving this session with or one skill you want to practice*.” “*Thanks for your active participation!”*

Once back in large groups, your recorder will be asked to ***briefly*** share the focus, insights and takeaways from their small group work. Record any particularly good quotes from your students to share with Nan and Alison.
