# Supplementary material for: What to Say When It Matters: Communication Skills to Address Implicit Bias Workshop
Source: MedEdPORTAL. 2025 Apr 15;21:11514. doi: 10.15766/mep_2374-8265.11514 (PMC11997152; doi:10.15766/mep_2374-8265.11514)
Supplement: Supplementary file 1 — Description of Microaggressions Workshop.docxEmail Advertisement.docxSurvey.docxCofacilitator Guide.docxLarge-Group Presentation.pptxGender Bias Simulation.mp4Student in Wheelchair Simulation.mp4Nursing Student Simulation.mp4Skills Card.docxMicroaggression Examples.docx [file mep_2374-8265.11514-s001.zip › E. Large-Group Presentation.pptx]

## Slide 1
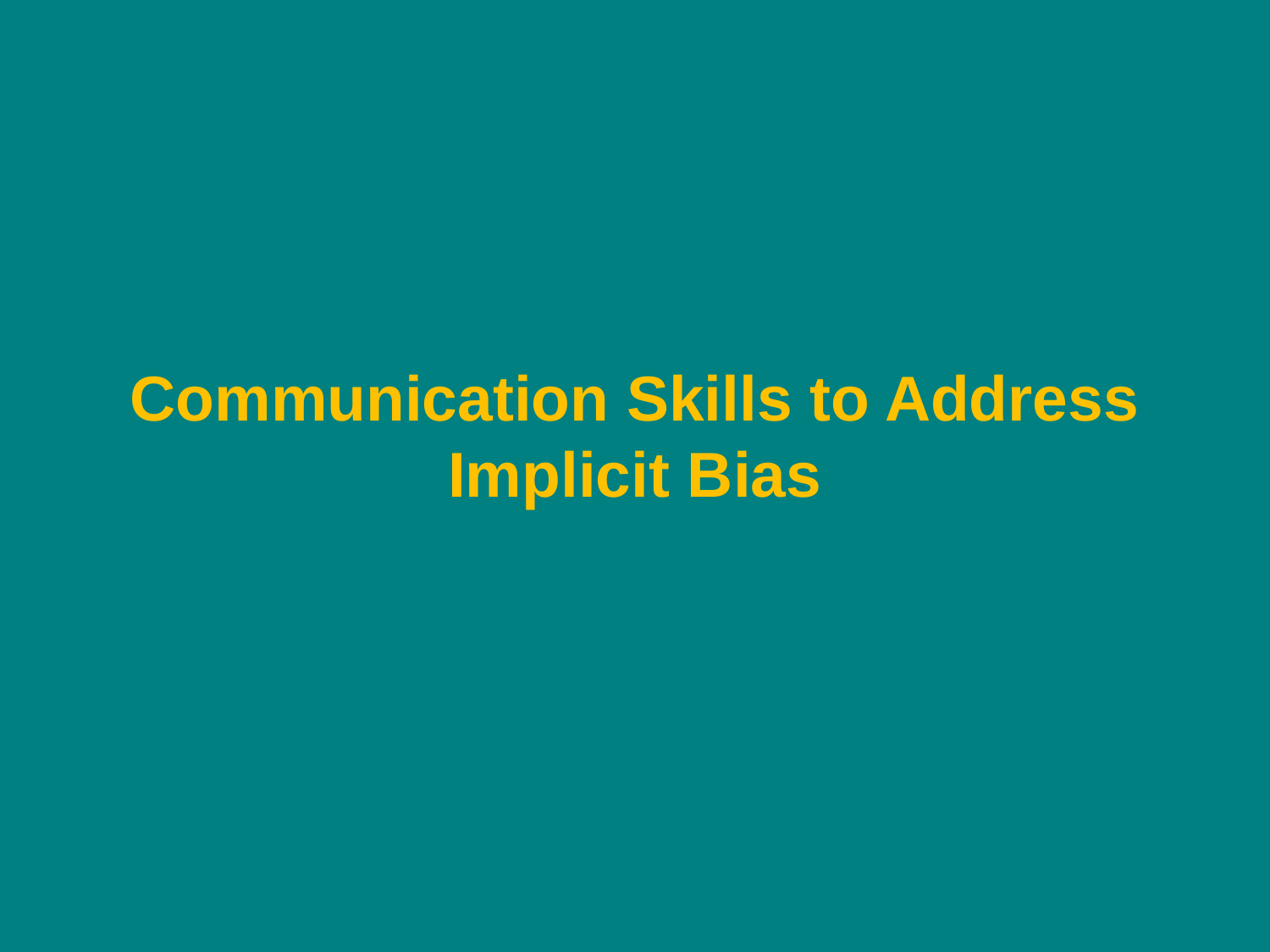

# Communication Skills to Address Implicit Bias

## Slide 2
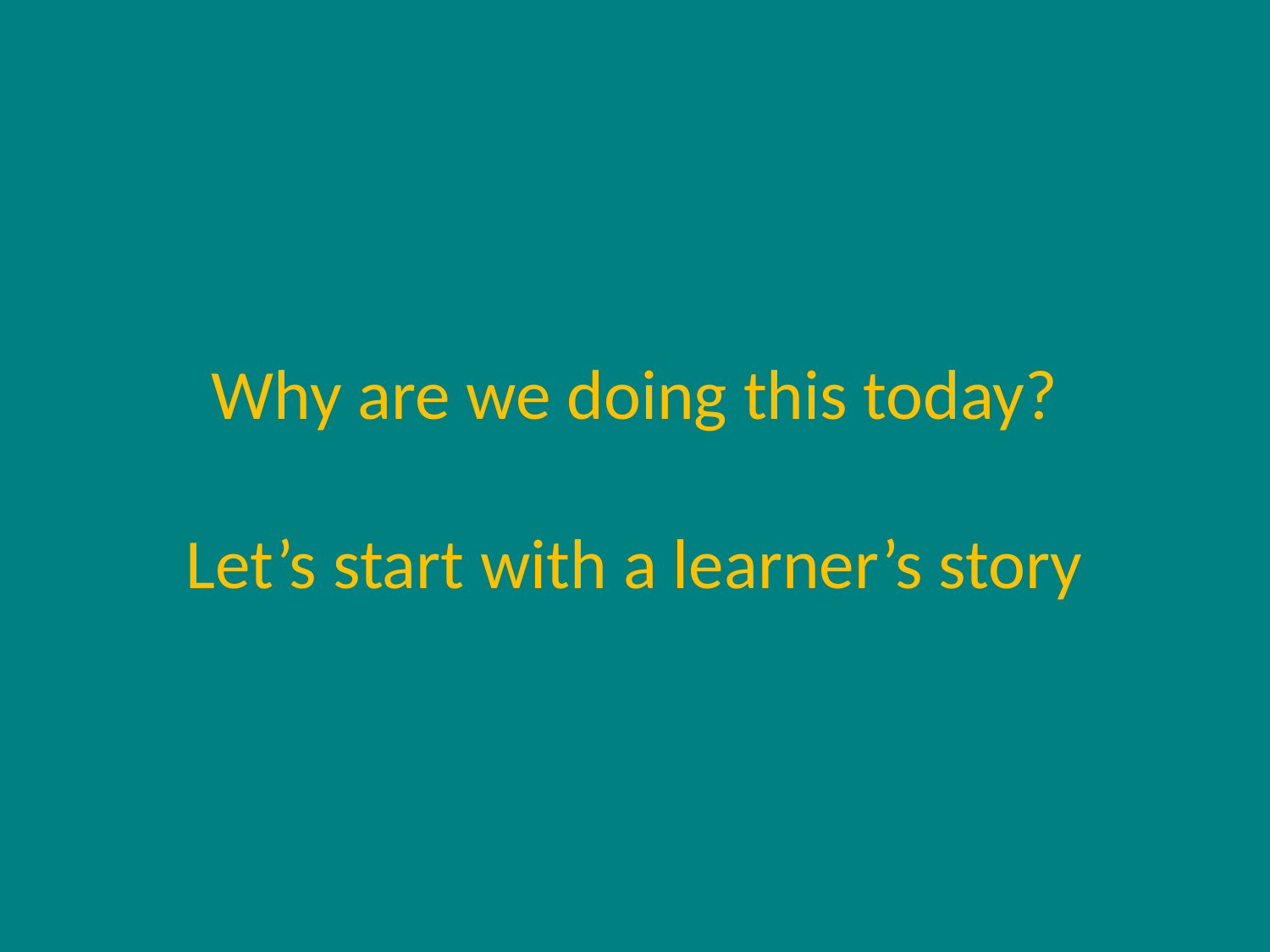

# Why are we doing this today?
Let’s start with a learner’s story

## Slide 3
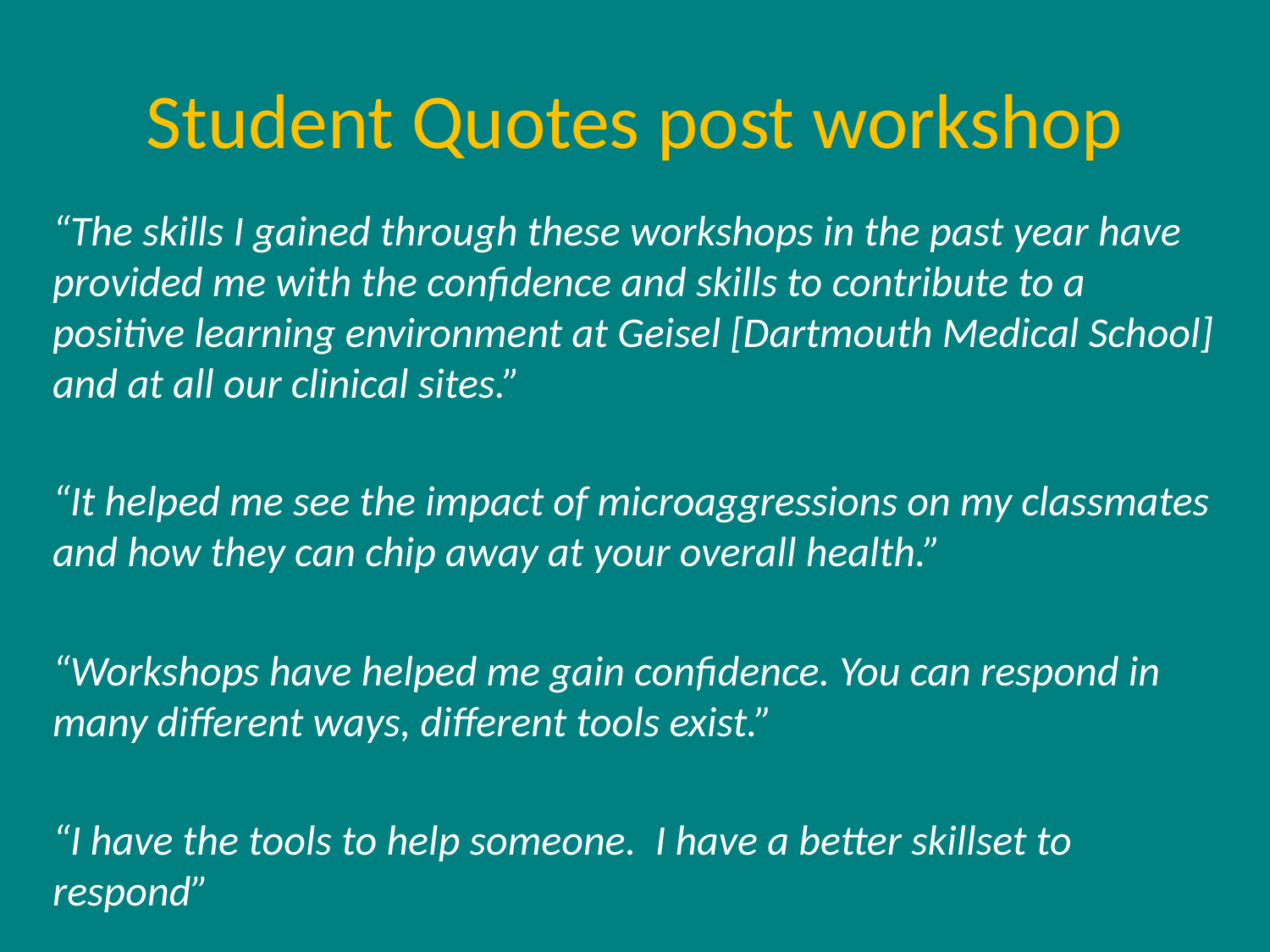

# Student Quotes post workshop
“The skills I gained through these workshops in the past year have provided me with the confidence and skills to contribute to a positive learning environment at Geisel [Dartmouth Medical School] and at all our clinical sites.”
“It helped me see the impact of microaggressions on my classmates and how they can chip away at your overall health.”
“Workshops have helped me gain confidence. You can respond in many different ways, different tools exist.”
“I have the tools to help someone. I have a better skillset to respond”

## Slide 4
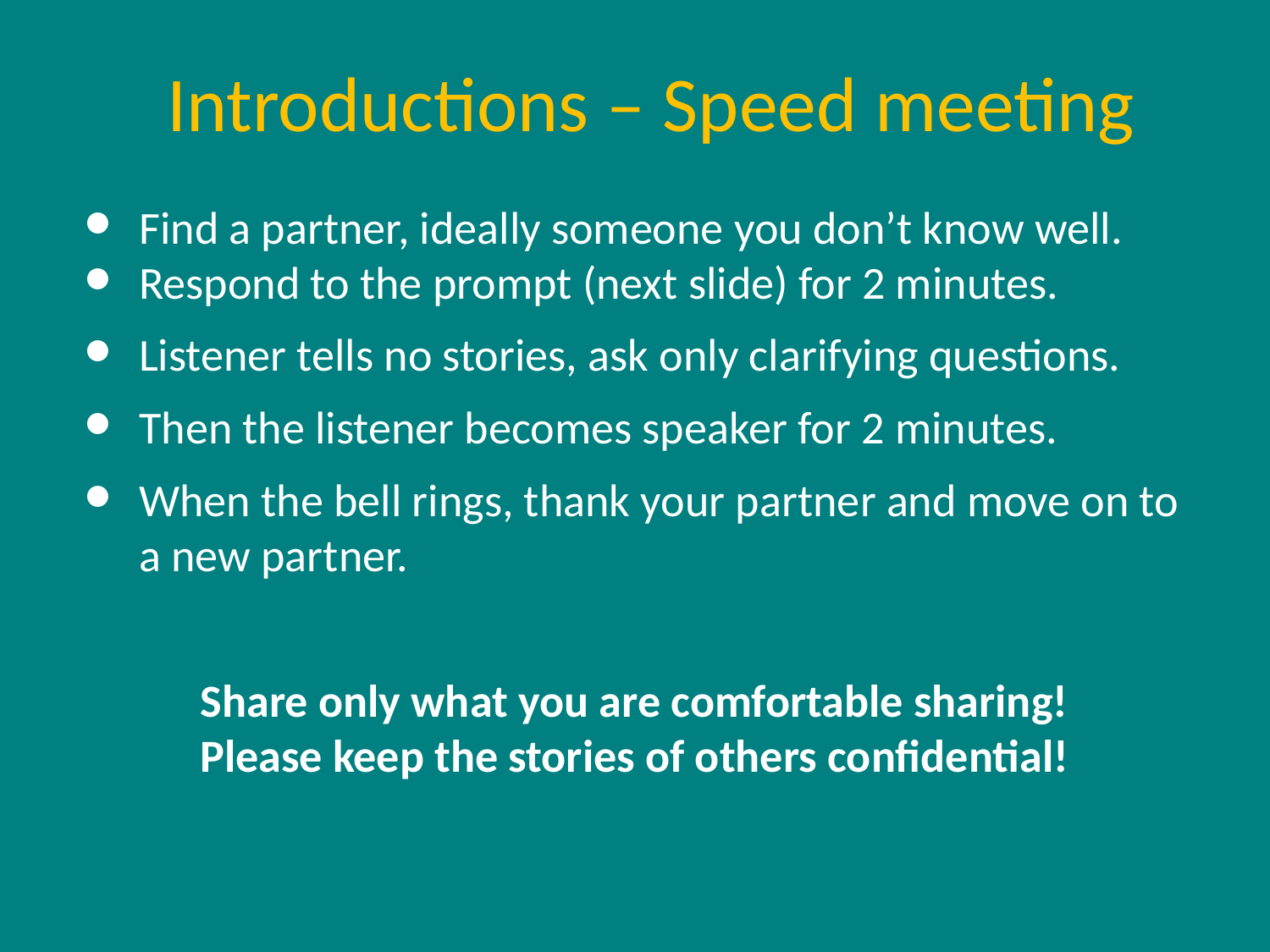

# Introductions – Speed meeting
Find a partner, ideally someone you don’t know well.
Respond to the prompt (next slide) for 2 minutes.
Listener tells no stories, ask only clarifying questions.
Then the listener becomes speaker for 2 minutes.
When the bell rings, thank your partner and move on to a new partner.
Share only what you are comfortable sharing!
Please keep the stories of others confidential!

## Slide 5
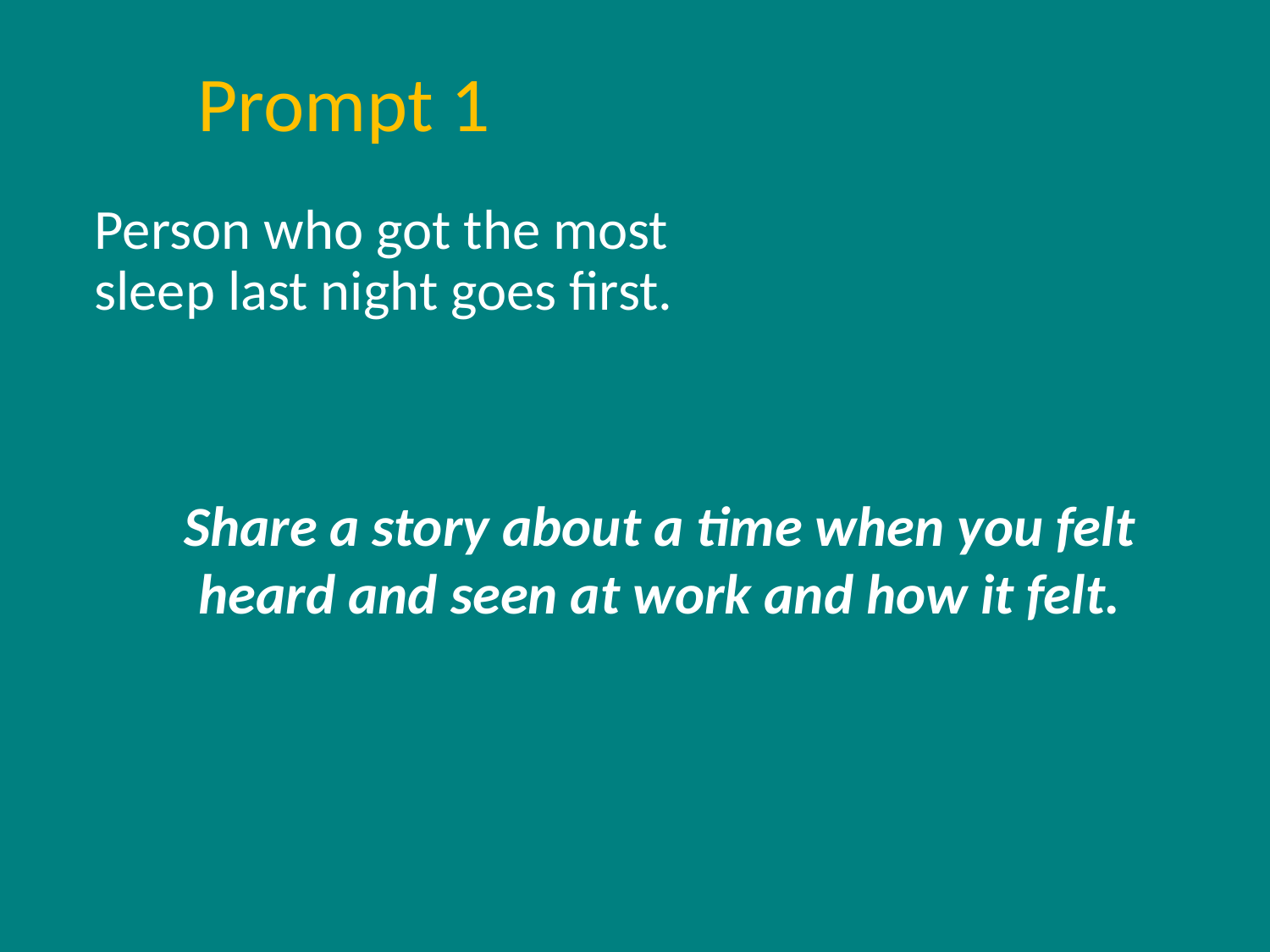

# Prompt 1
Person who got the most sleep last night goes first.
Share a story about a time when you felt heard and seen at work and how it felt.

## Slide 6
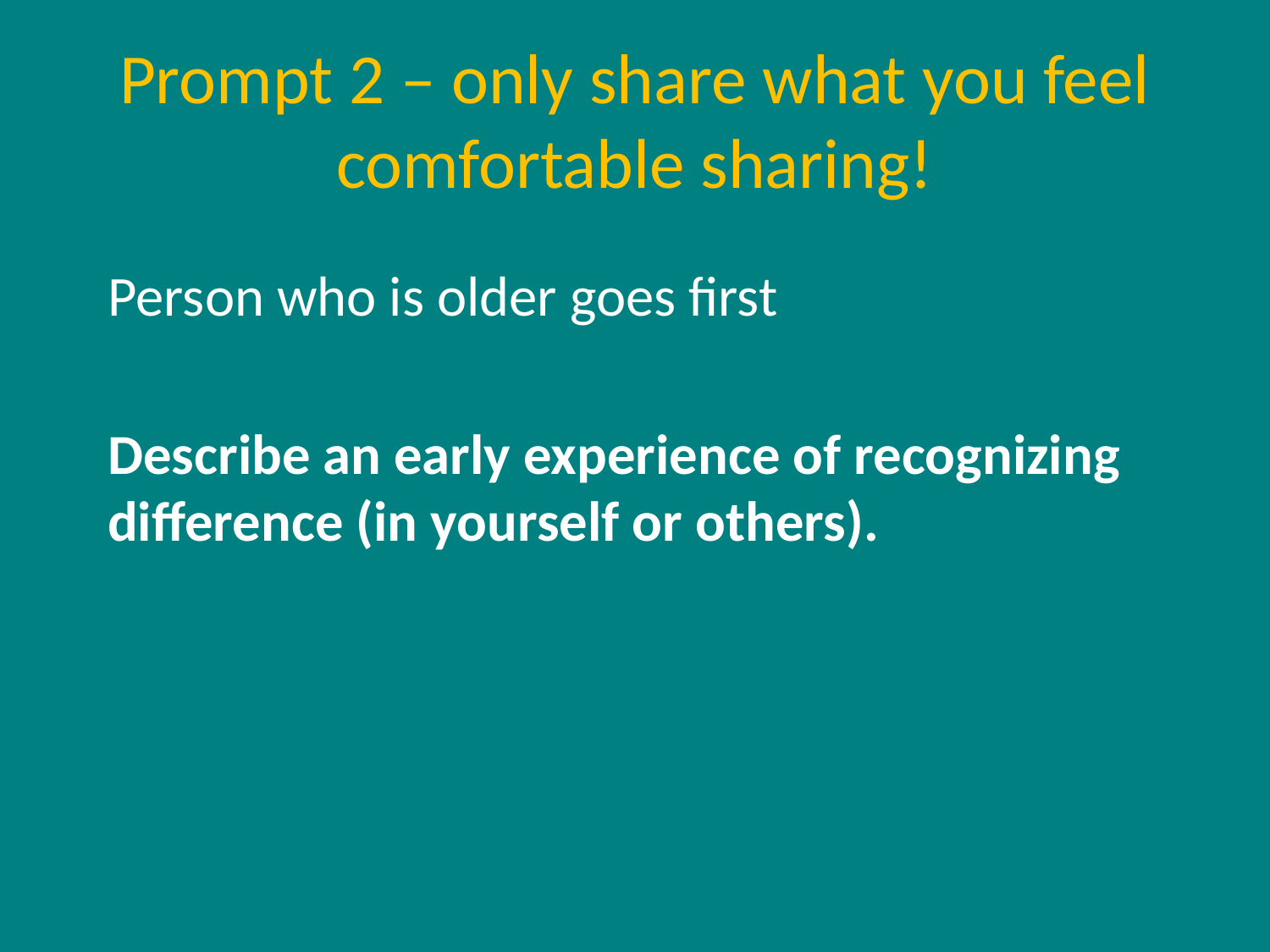

# Prompt 2 – only share what you feel comfortable sharing!
Person who is older goes first
Describe an early experience of recognizing difference (in yourself or others).

## Slide 7
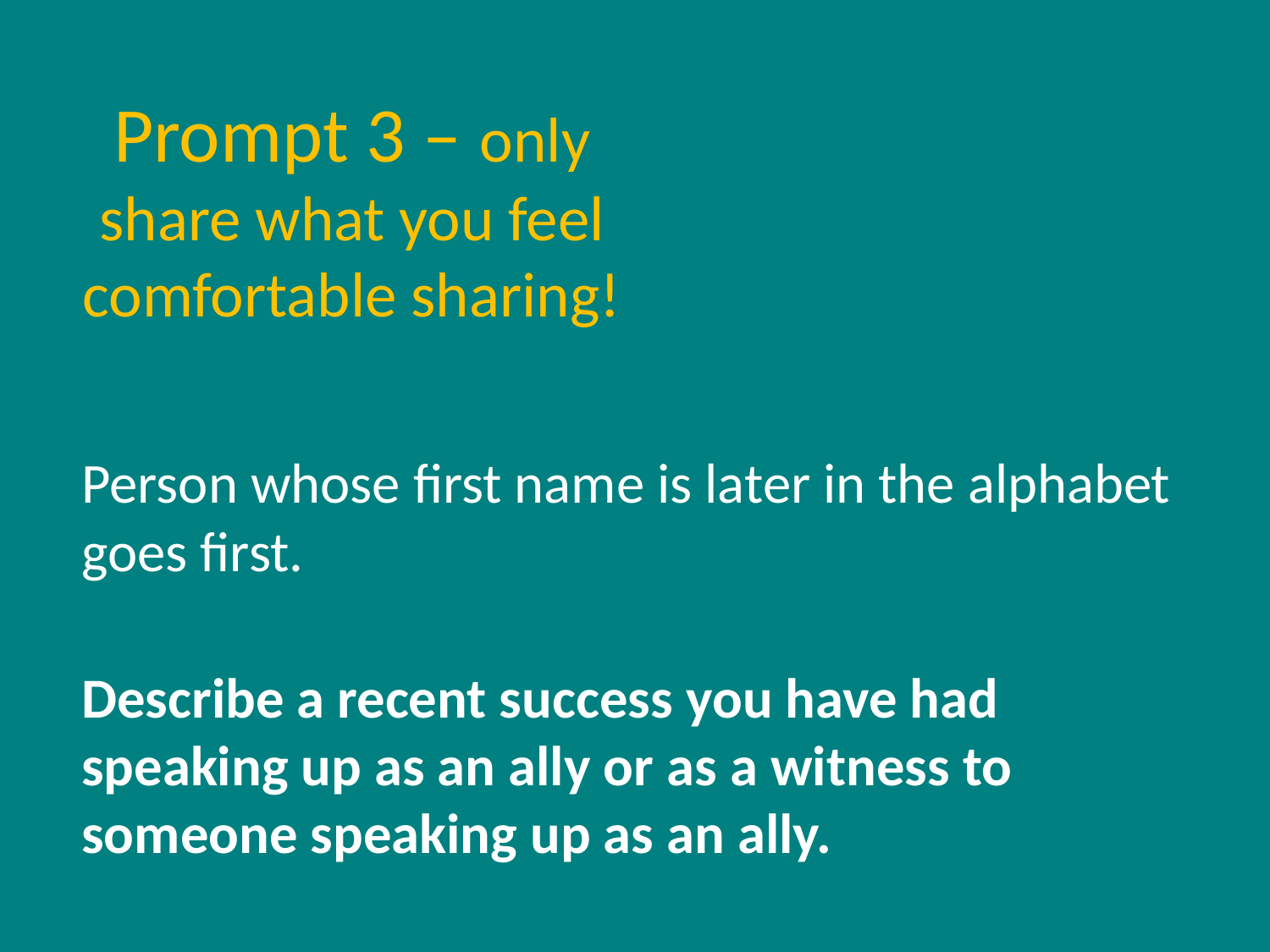

# Prompt 3 – only share what you feel comfortable sharing!
Person whose first name is later in the alphabet goes first.
Describe a recent success you have had speaking up as an ally or as a witness to someone speaking up as an ally.

## Slide 8
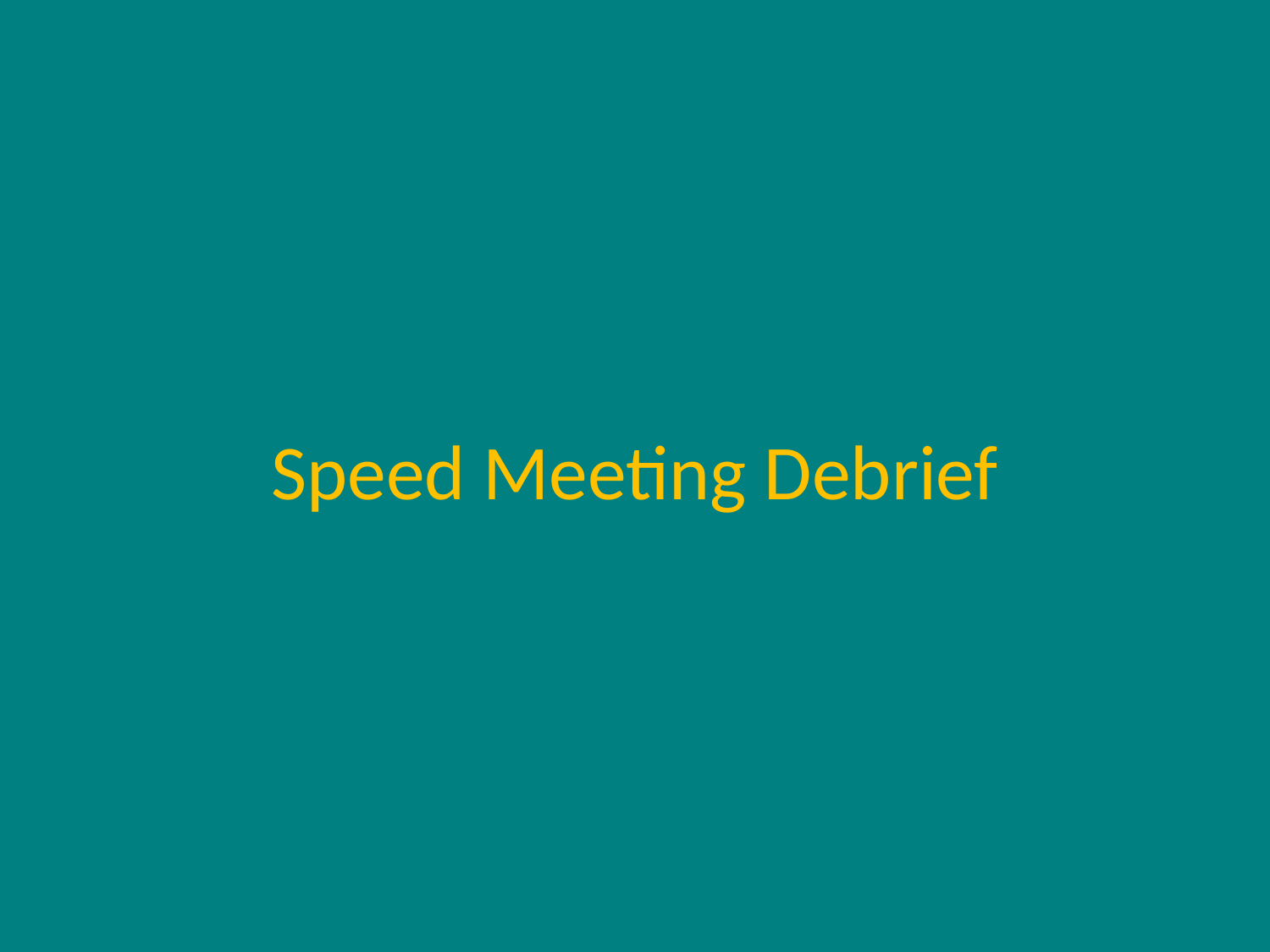

# Speed Meeting Debrief

## Slide 9
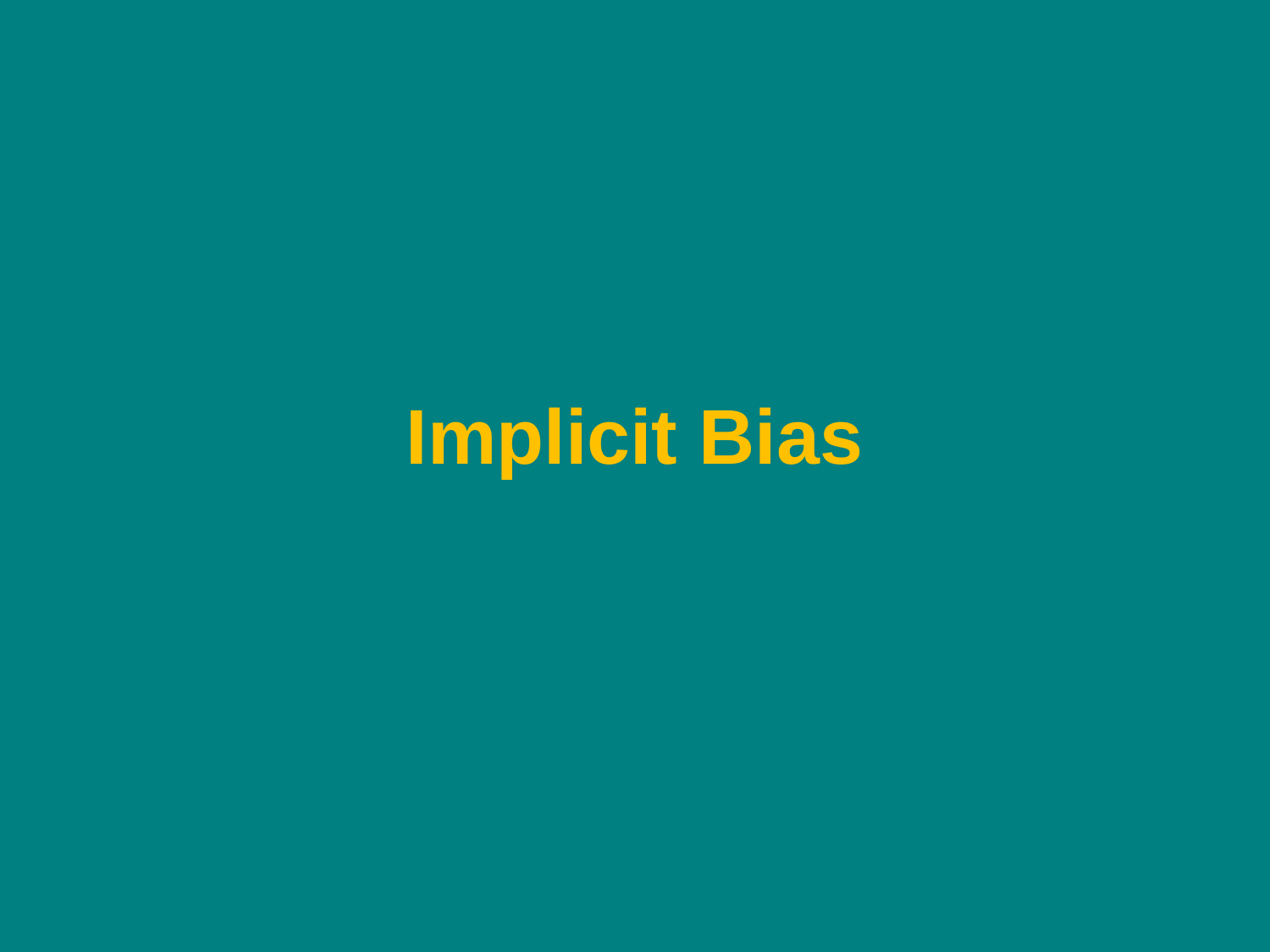

# Implicit Bias

## Slide 10
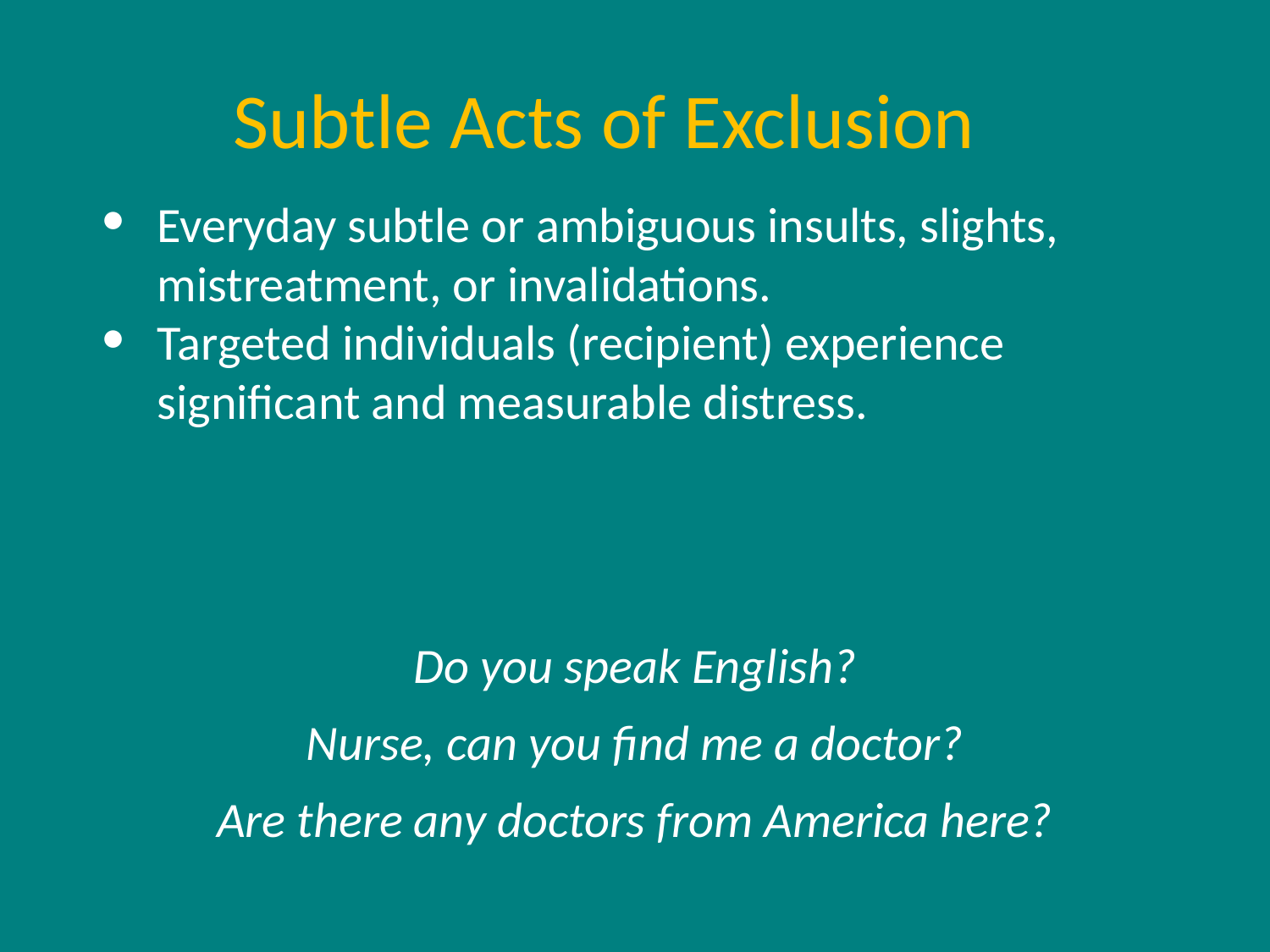

# Subtle Acts of Exclusion
Everyday subtle or ambiguous insults, slights, mistreatment, or invalidations.
Targeted individuals (recipient) experience significant and measurable distress.
Do you speak English?
Nurse, can you find me a doctor?
Are there any doctors from America here?

## Slide 11
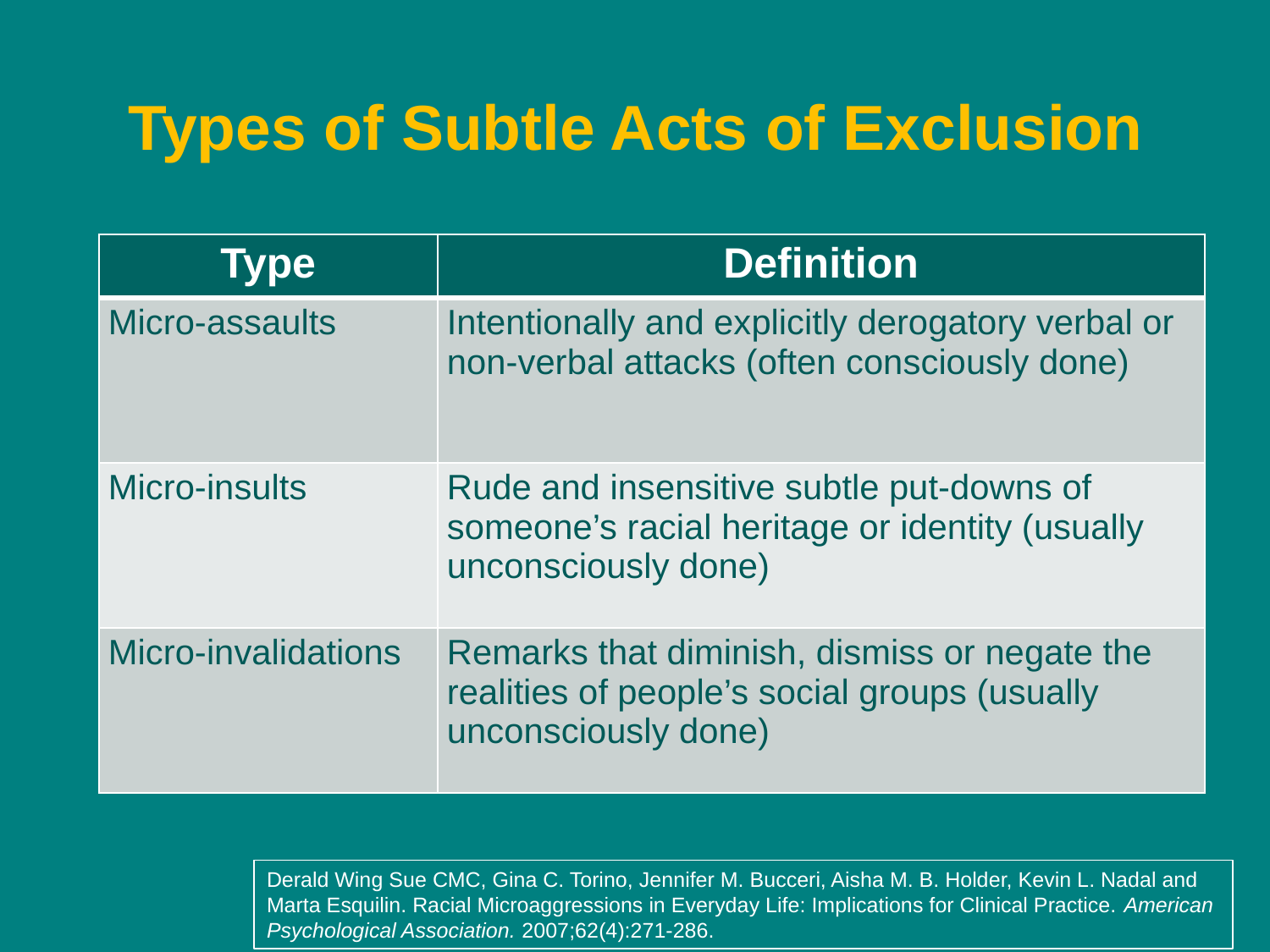

# Types of Subtle Acts of Exclusion
| Type | Definition |
| --- | --- |
| Micro-assaults | Intentionally and explicitly derogatory verbal or non-verbal attacks (often consciously done) |
| Micro-insults | Rude and insensitive subtle put-downs of someone’s racial heritage or identity (usually unconsciously done) |
| Micro-invalidations | Remarks that diminish, dismiss or negate the realities of people’s social groups (usually unconsciously done) |
Center for a Diverse Healthcare Workforce
Derald Wing Sue CMC, Gina C. Torino, Jennifer M. Bucceri, Aisha M. B. Holder, Kevin L. Nadal and Marta Esquilin. Racial Microaggressions in Everyday Life: Implications for Clinical Practice. American Psychological Association. 2007;62(4):271-286.

## Slide 12
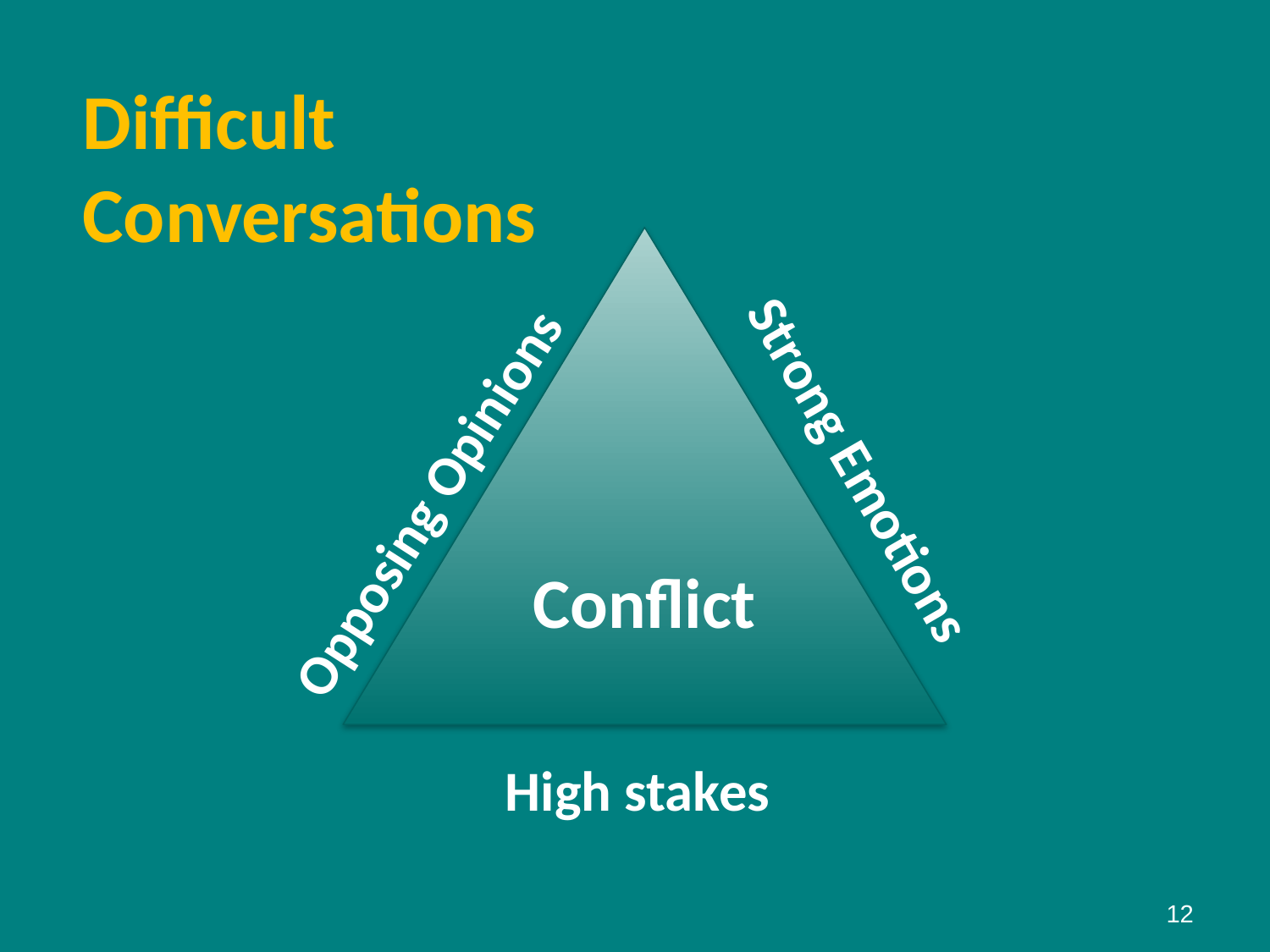

Difficult Conversations
Conflict
Opposing Opinions
Strong Emotions
High stakes
‹#›

## Slide 13
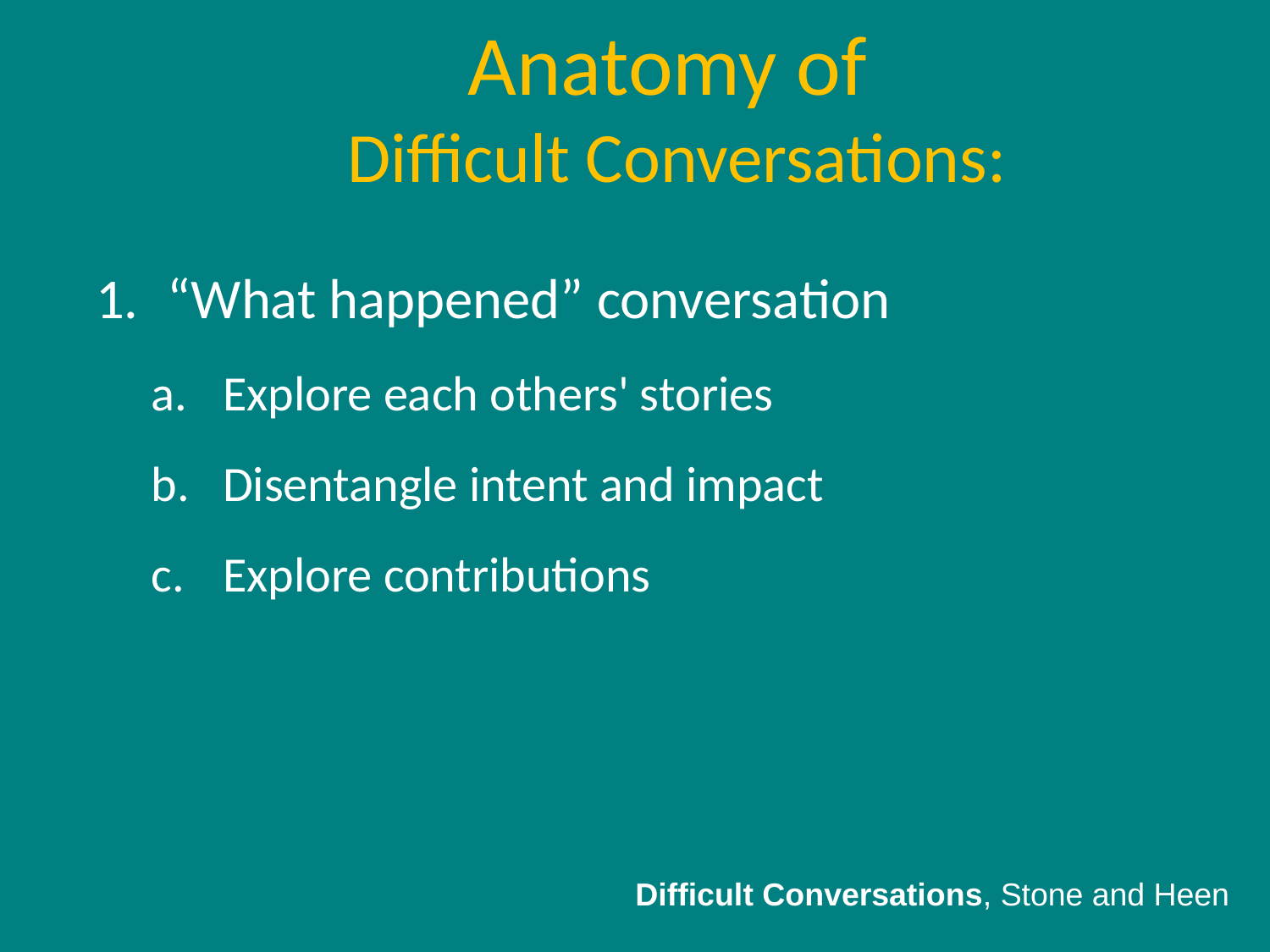

# Anatomy of Difficult Conversations:
“What happened” conversation
Explore each others' stories
Disentangle intent and impact
Explore contributions
Difficult Conversations, Stone and Heen

## Slide 14
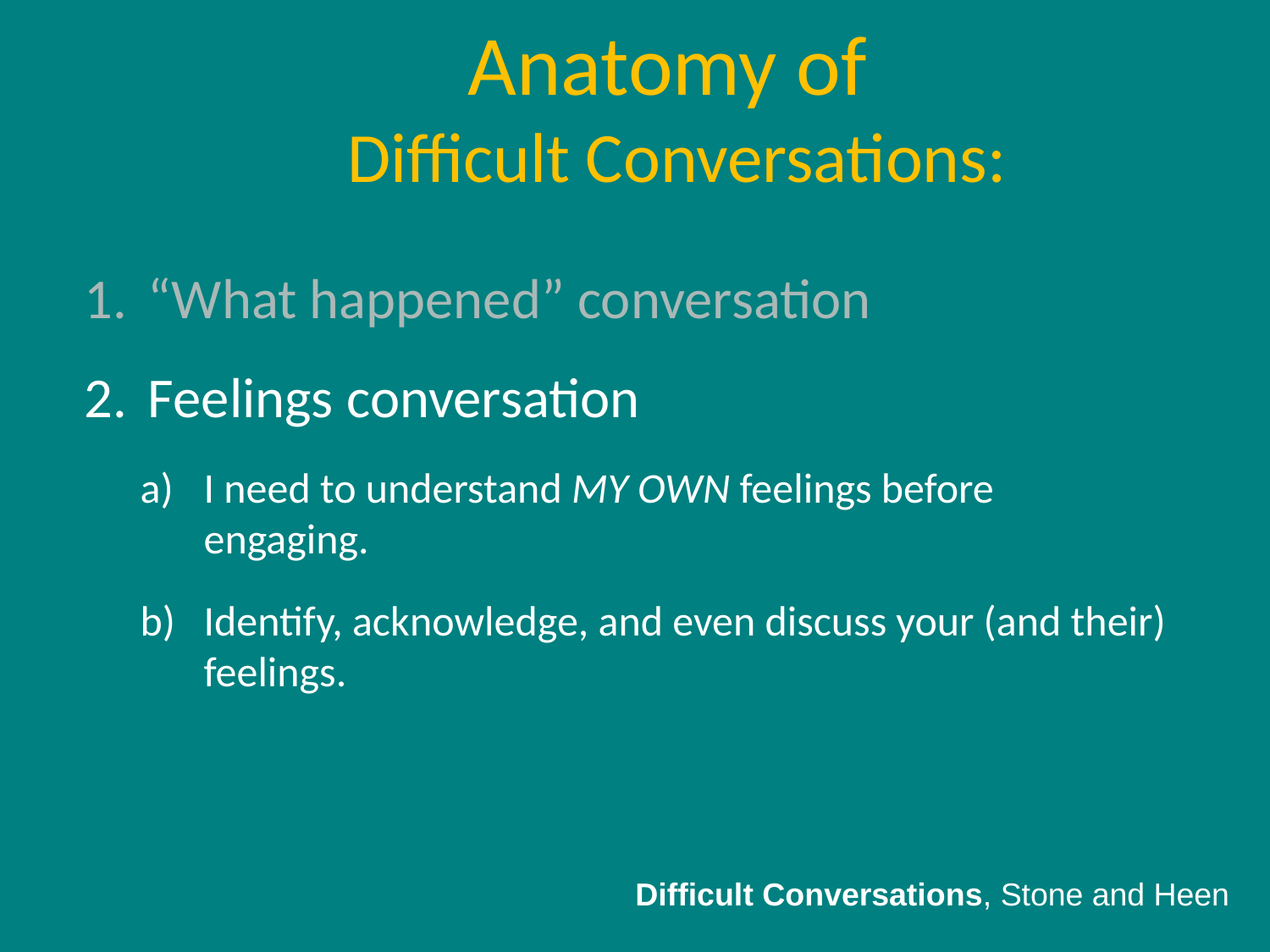

# Anatomy of Difficult Conversations:
“What happened” conversation
Feelings conversation
I need to understand MY OWN feelings before engaging.
Identify, acknowledge, and even discuss your (and their) feelings.
Difficult Conversations, Stone and Heen

## Slide 15
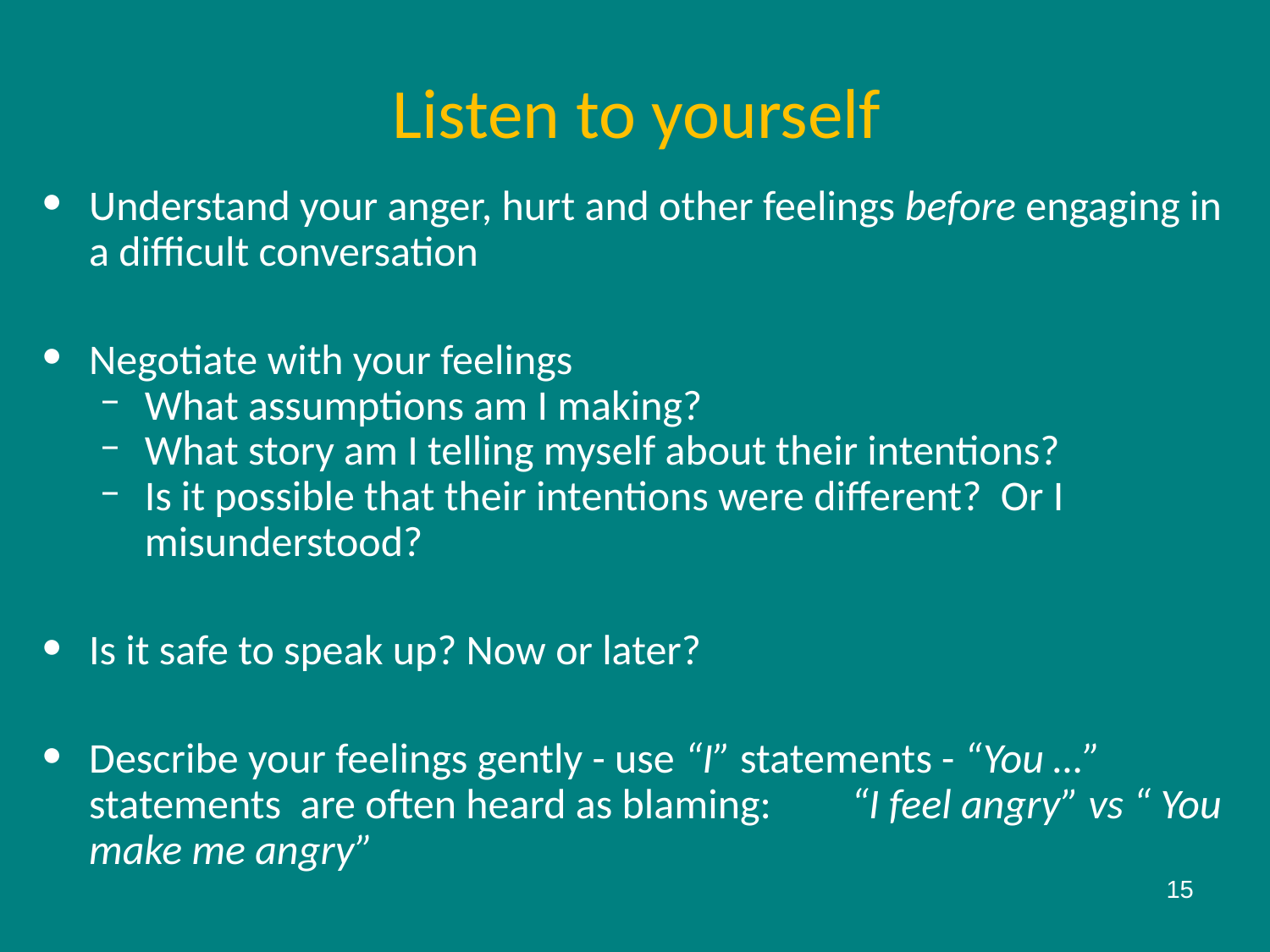

# Listen to yourself
Understand your anger, hurt and other feelings before engaging in a difficult conversation
Negotiate with your feelings
What assumptions am I making?
What story am I telling myself about their intentions?
Is it possible that their intentions were different? Or I misunderstood?
Is it safe to speak up? Now or later?
Describe your feelings gently - use “I” statements - “You …” statements are often heard as blaming:	“I feel angry” vs “ You make me angry”
‹#›

## Slide 16
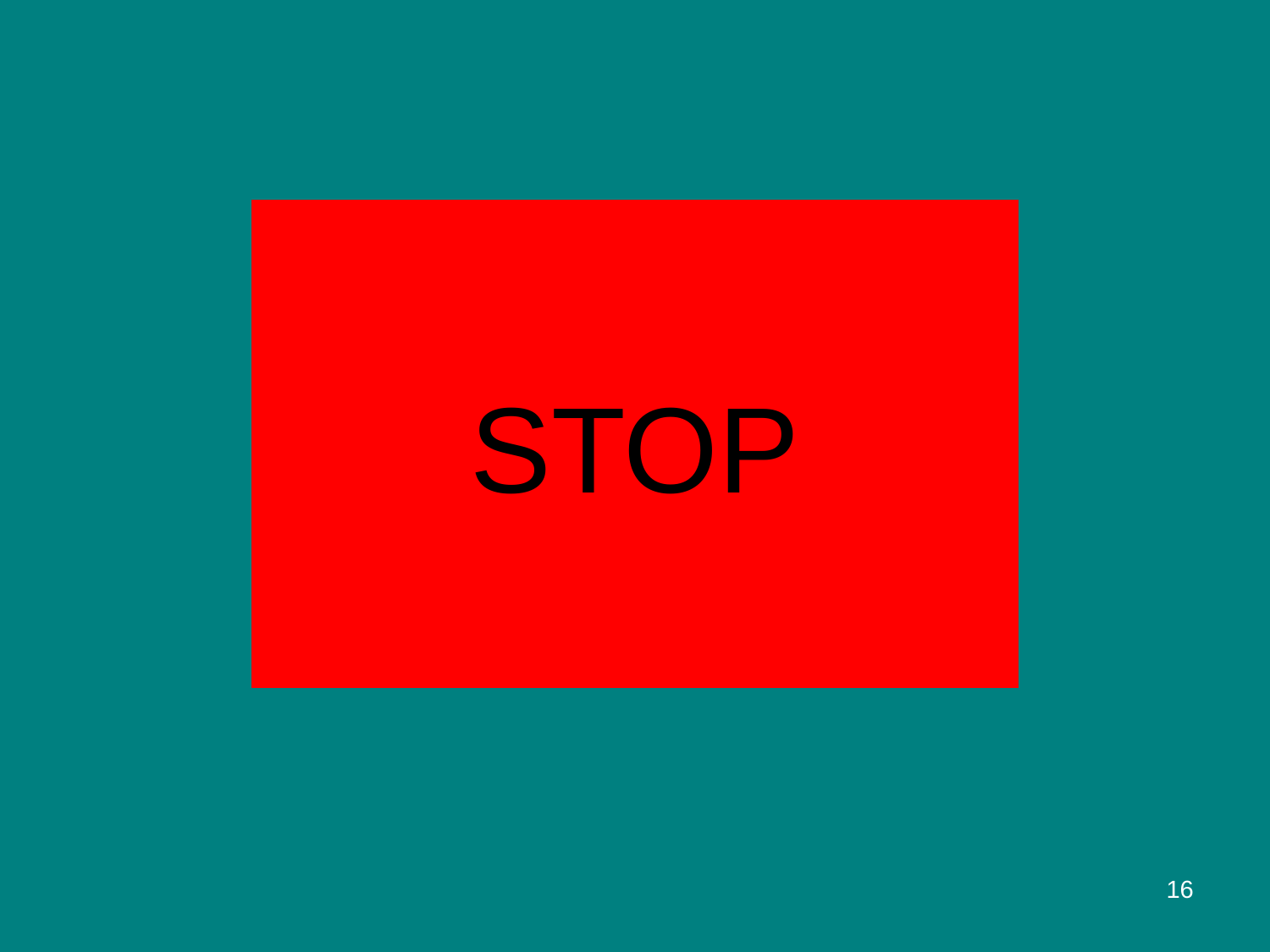

STOP
‹#›

## Slide 17
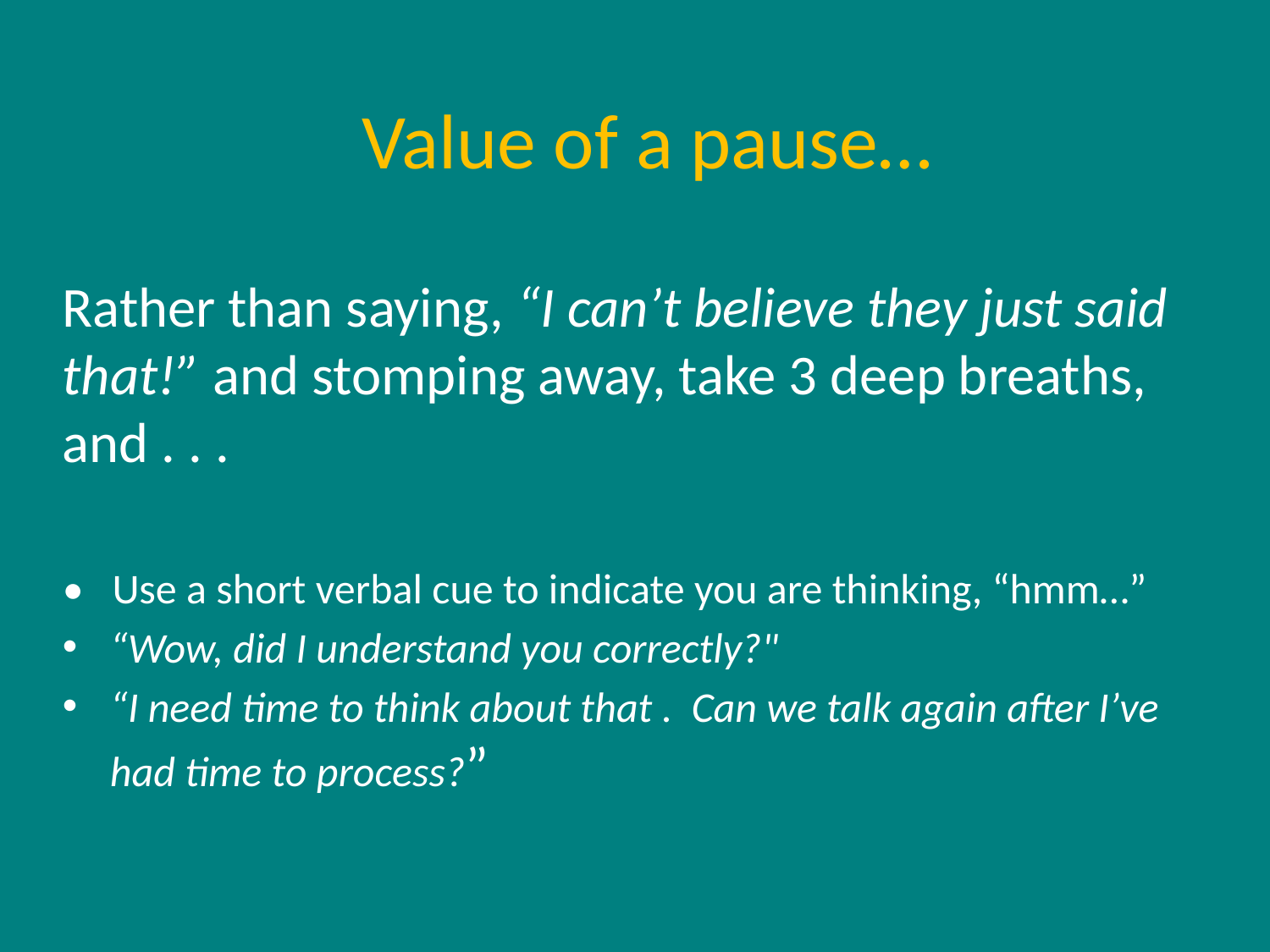

# Value of a pause…
Rather than saying, “I can’t believe they just said that!” and stomping away, take 3 deep breaths, and . . .
• Use a short verbal cue to indicate you are thinking, “hmm…”
“Wow, did I understand you correctly?"
“I need time to think about that . Can we talk again after I’ve had time to process?”

## Slide 18
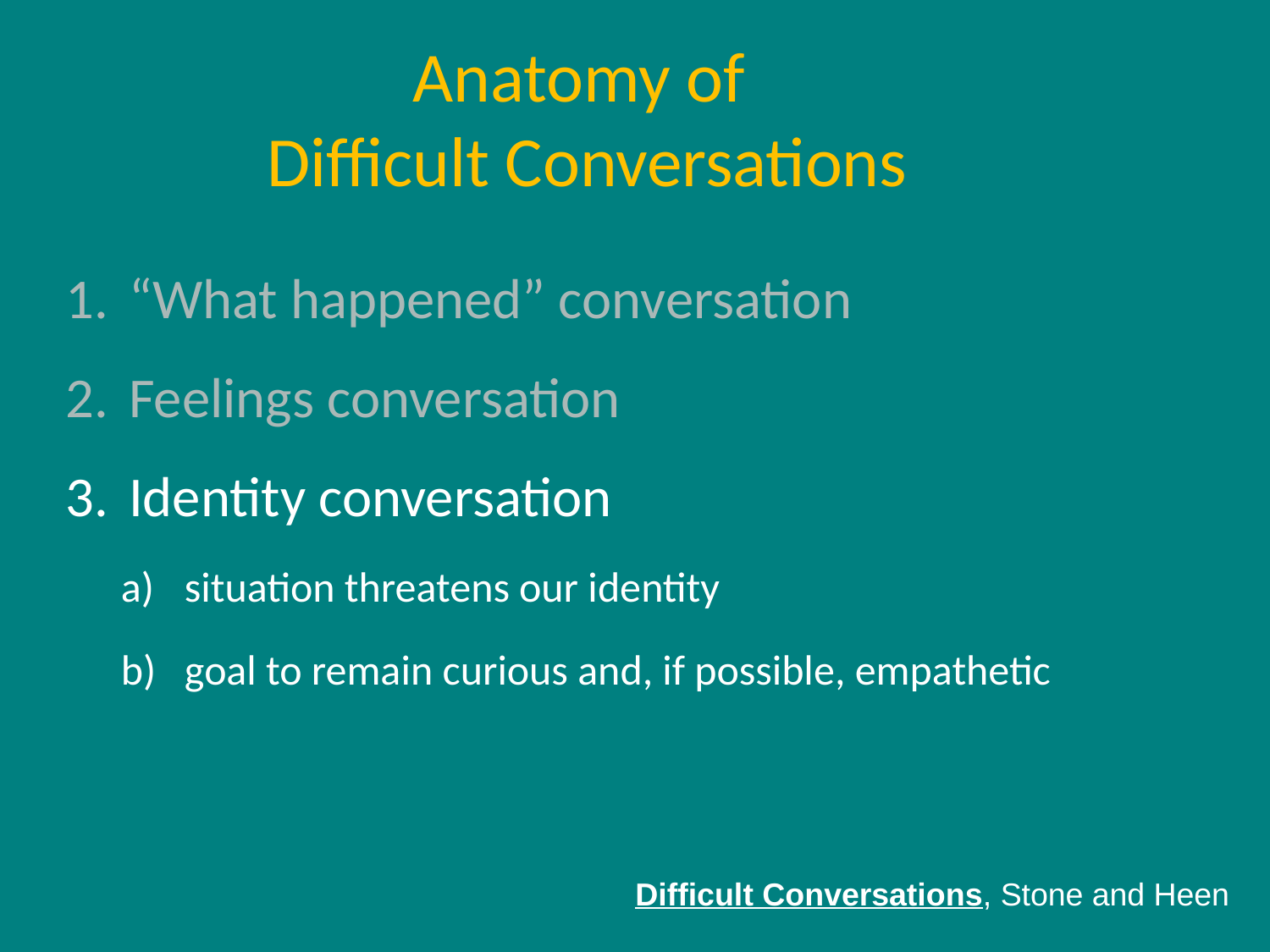

# Anatomy of Difficult Conversations
“What happened” conversation
Feelings conversation
Identity conversation
situation threatens our identity
goal to remain curious and, if possible, empathetic
Difficult Conversations, Stone and Heen

## Slide 19
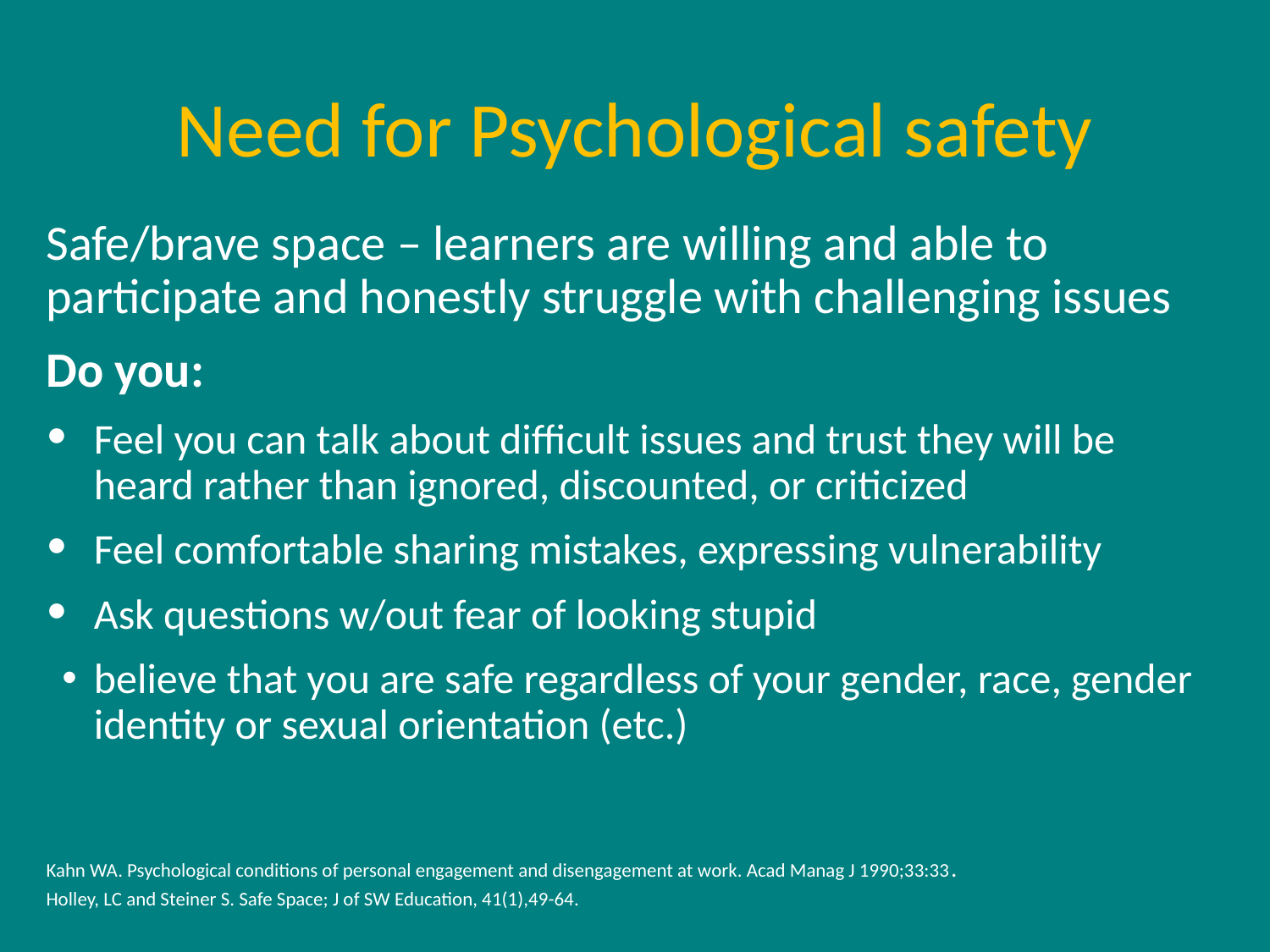

# Need for Psychological safety
Safe/brave space – learners are willing and able to participate and honestly struggle with challenging issues
Do you:
Feel you can talk about difficult issues and trust they will be heard rather than ignored, discounted, or criticized
Feel comfortable sharing mistakes, expressing vulnerability
Ask questions w/out fear of looking stupid
believe that you are safe regardless of your gender, race, gender identity or sexual orientation (etc.)
Kahn WA. Psychological conditions of personal engagement and disengagement at work. Acad Manag J 1990;33:33.
Holley, LC and Steiner S. Safe Space; J of SW Education, 41(1),49-64.

## Slide 20
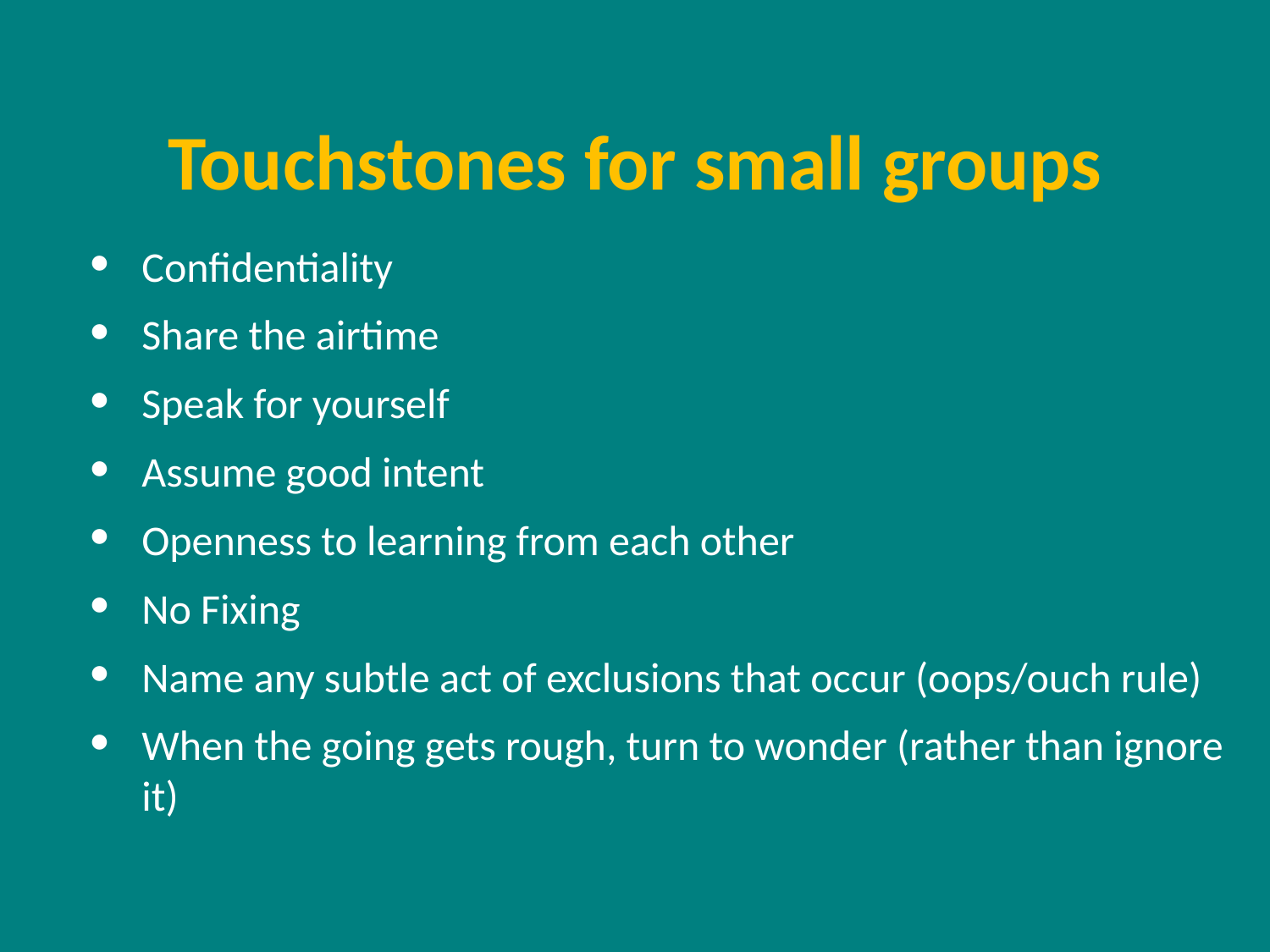

# Touchstones for small groups
Confidentiality
Share the airtime
Speak for yourself
Assume good intent
Openness to learning from each other
No Fixing
Name any subtle act of exclusions that occur (oops/ouch rule)
When the going gets rough, turn to wonder (rather than ignore it)

## Slide 21
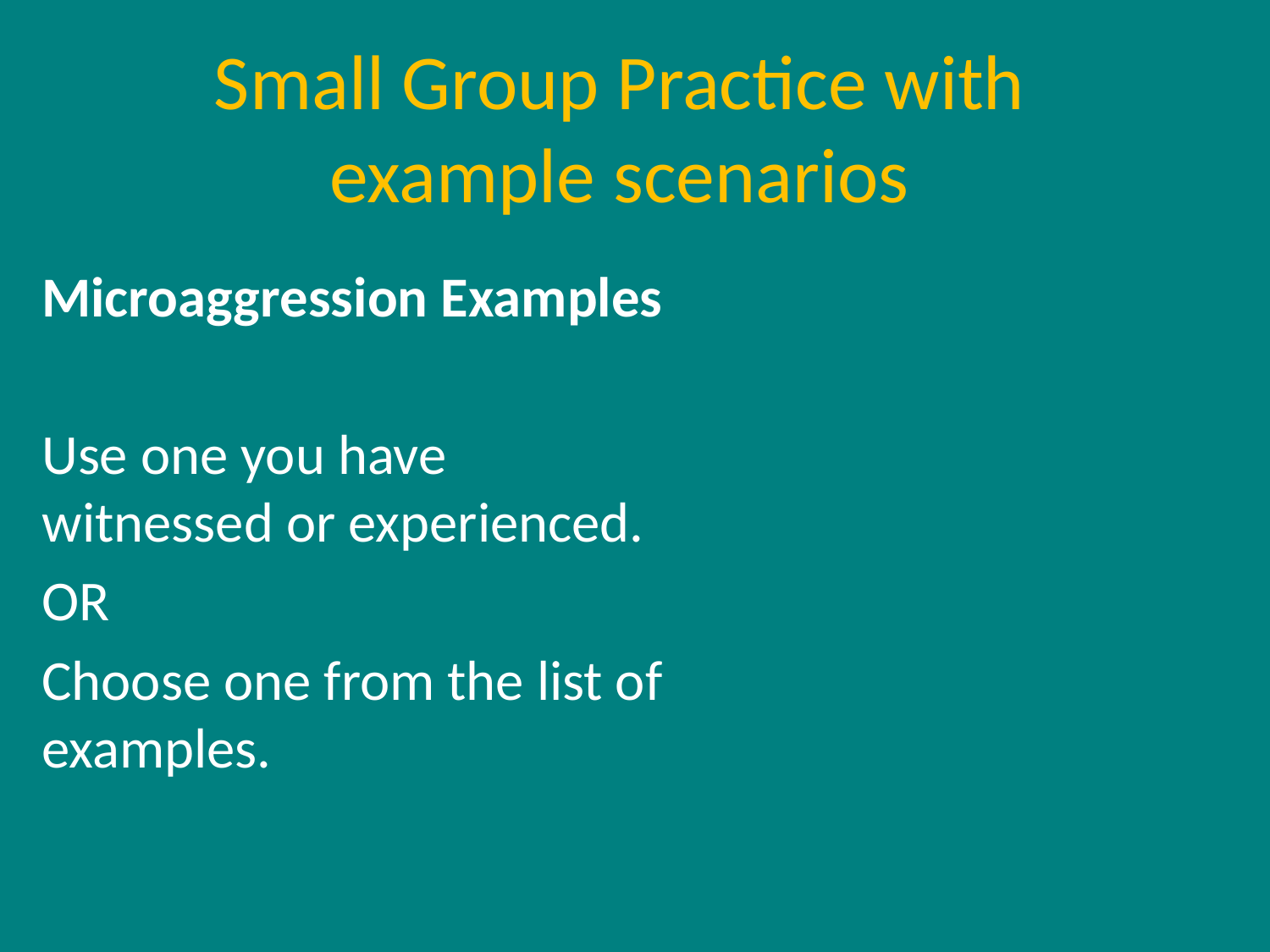

# Small Group Practice with example scenarios
Microaggression Examples
Use one you have witnessed or experienced.
OR
Choose one from the list of examples.

## Slide 22
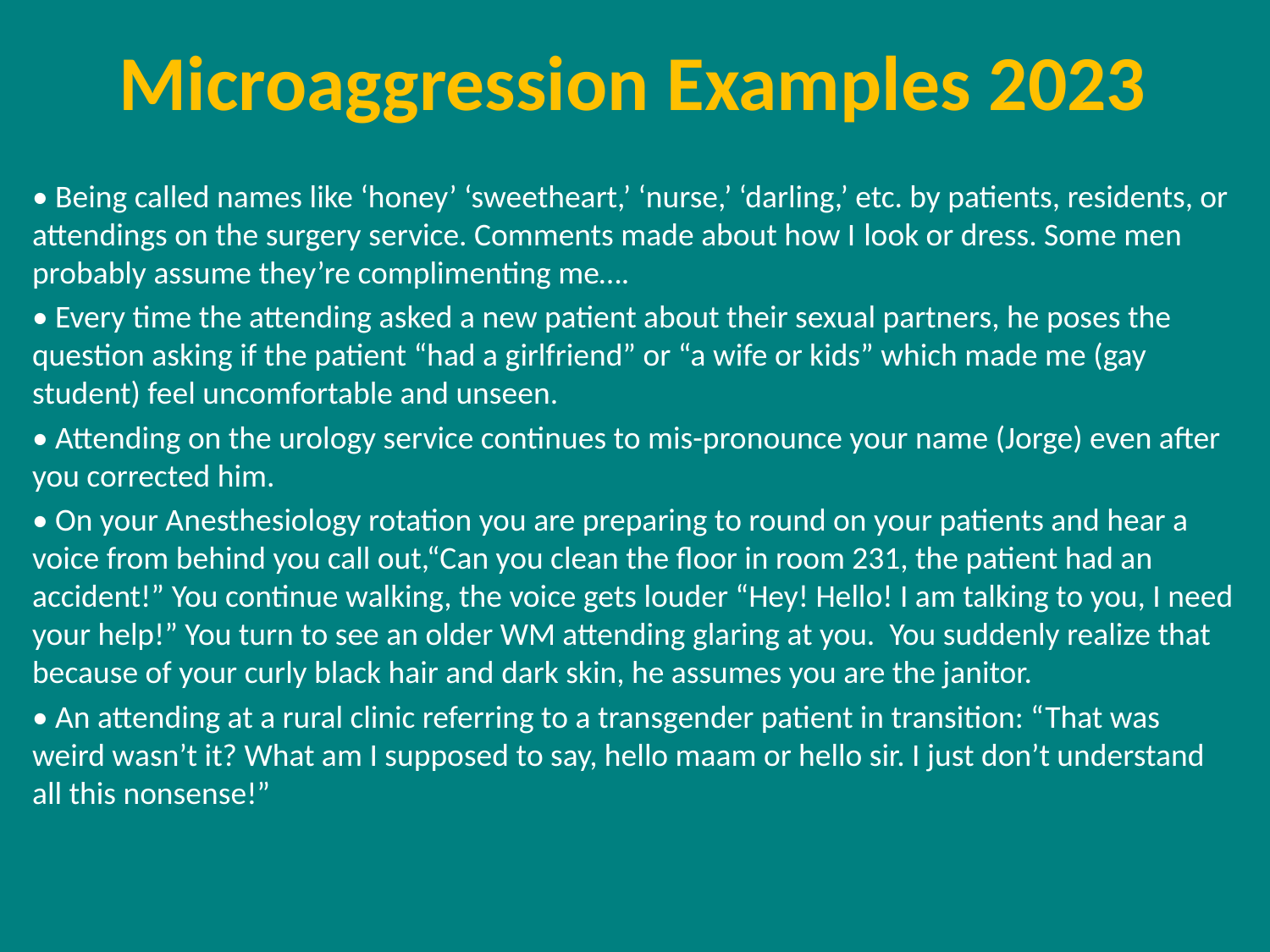

# Microaggression Examples 2023
• Being called names like ‘honey’ ‘sweetheart,’ ‘nurse,’ ‘darling,’ etc. by patients, residents, or attendings on the surgery service. Comments made about how I look or dress. Some men probably assume they’re complimenting me….
• Every time the attending asked a new patient about their sexual partners, he poses the question asking if the patient “had a girlfriend” or “a wife or kids” which made me (gay student) feel uncomfortable and unseen.
• Attending on the urology service continues to mis-pronounce your name (Jorge) even after you corrected him.
• On your Anesthesiology rotation you are preparing to round on your patients and hear a voice from behind you call out,“Can you clean the floor in room 231, the patient had an accident!” You continue walking, the voice gets louder “Hey! Hello! I am talking to you, I need your help!” You turn to see an older WM attending glaring at you. You suddenly realize that because of your curly black hair and dark skin, he assumes you are the janitor.
• An attending at a rural clinic referring to a transgender patient in transition: “That was weird wasn’t it? What am I supposed to say, hello maam or hello sir. I just don’t understand all this nonsense!”

## Slide 23
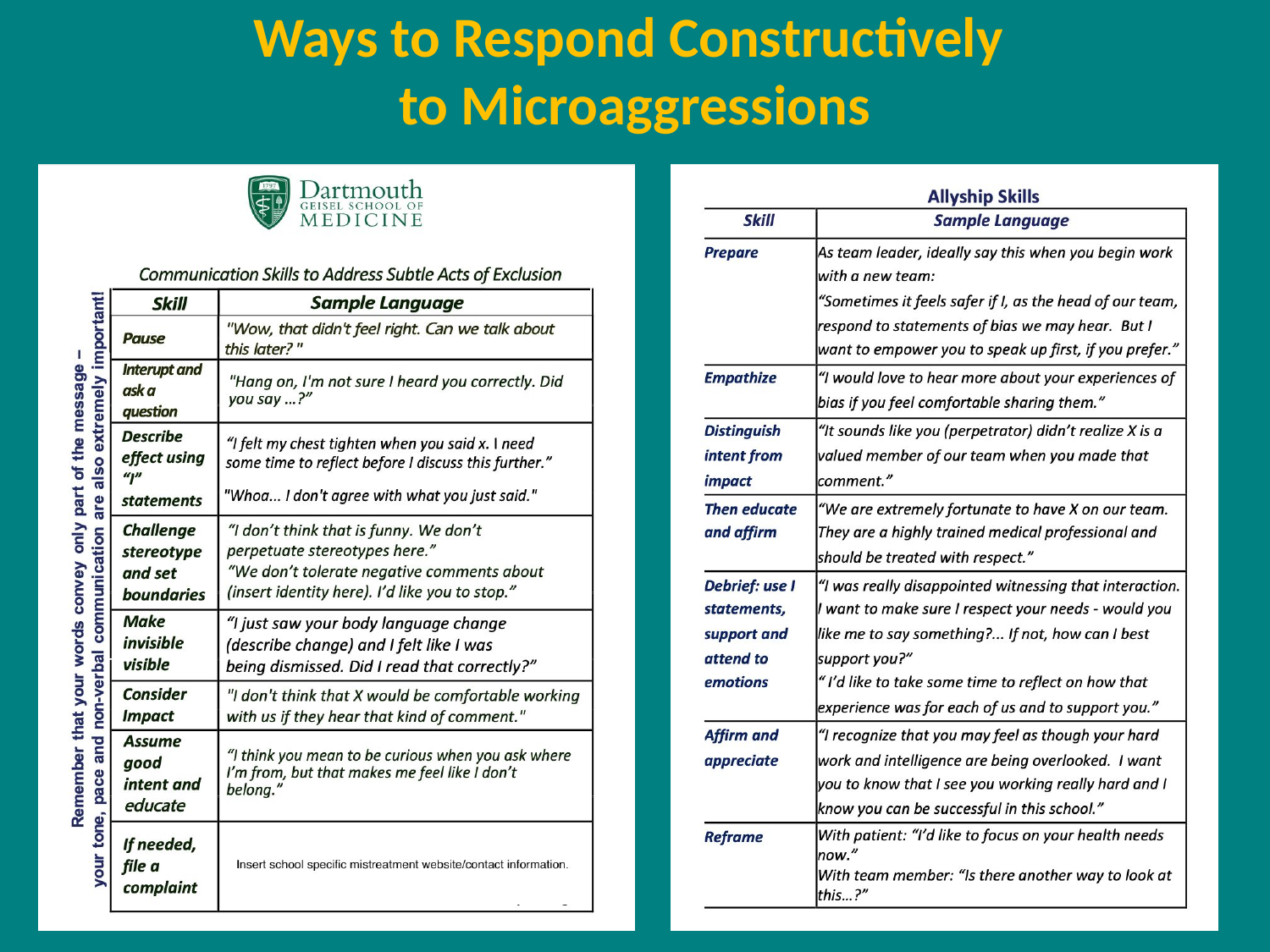

# Ways to Respond Constructively to Microaggressions

## Slide 24
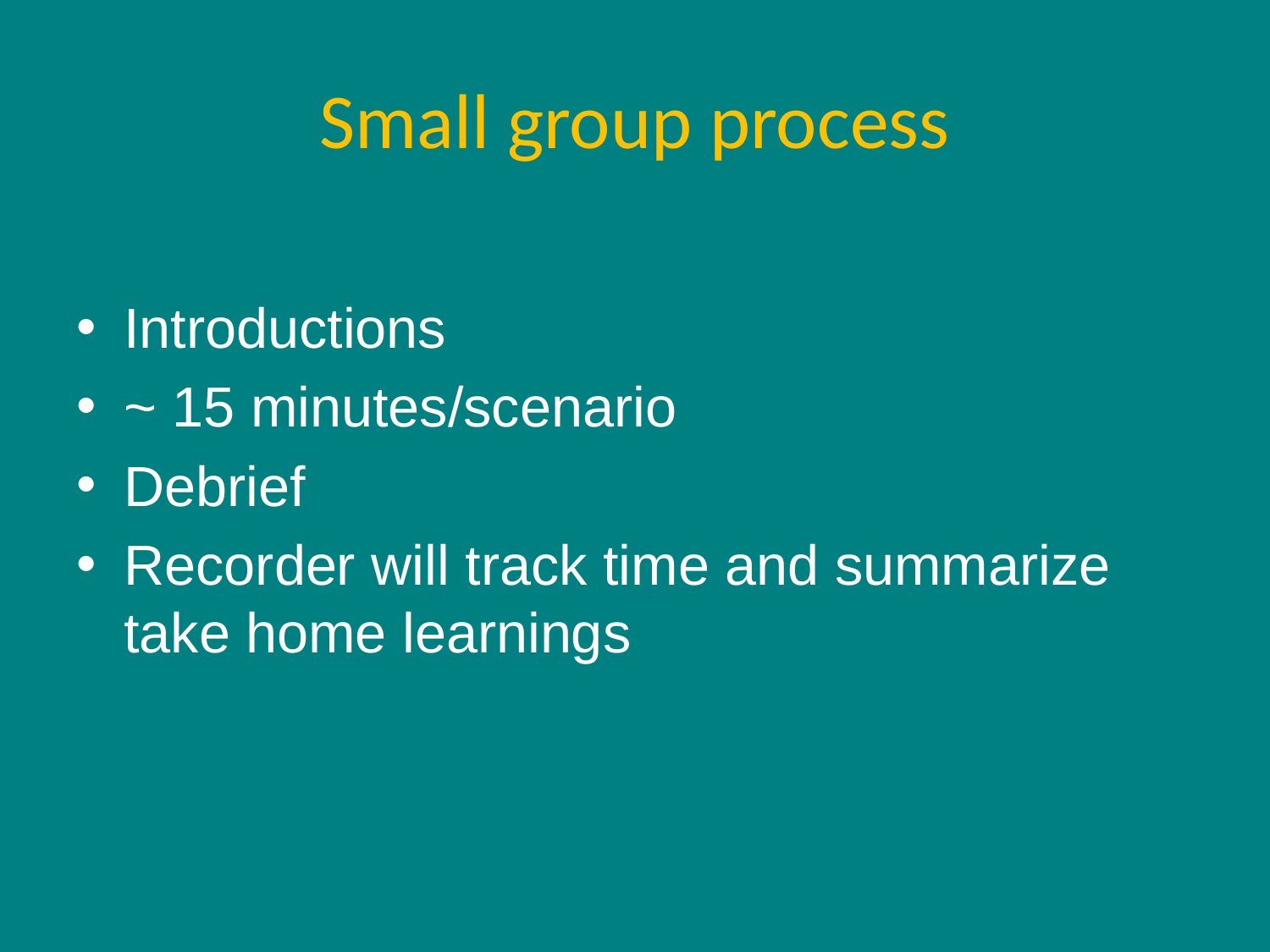

# Small group process
Introductions
~ 15 minutes/scenario
Debrief
Recorder will track time and summarize take home learnings

## Slide 25
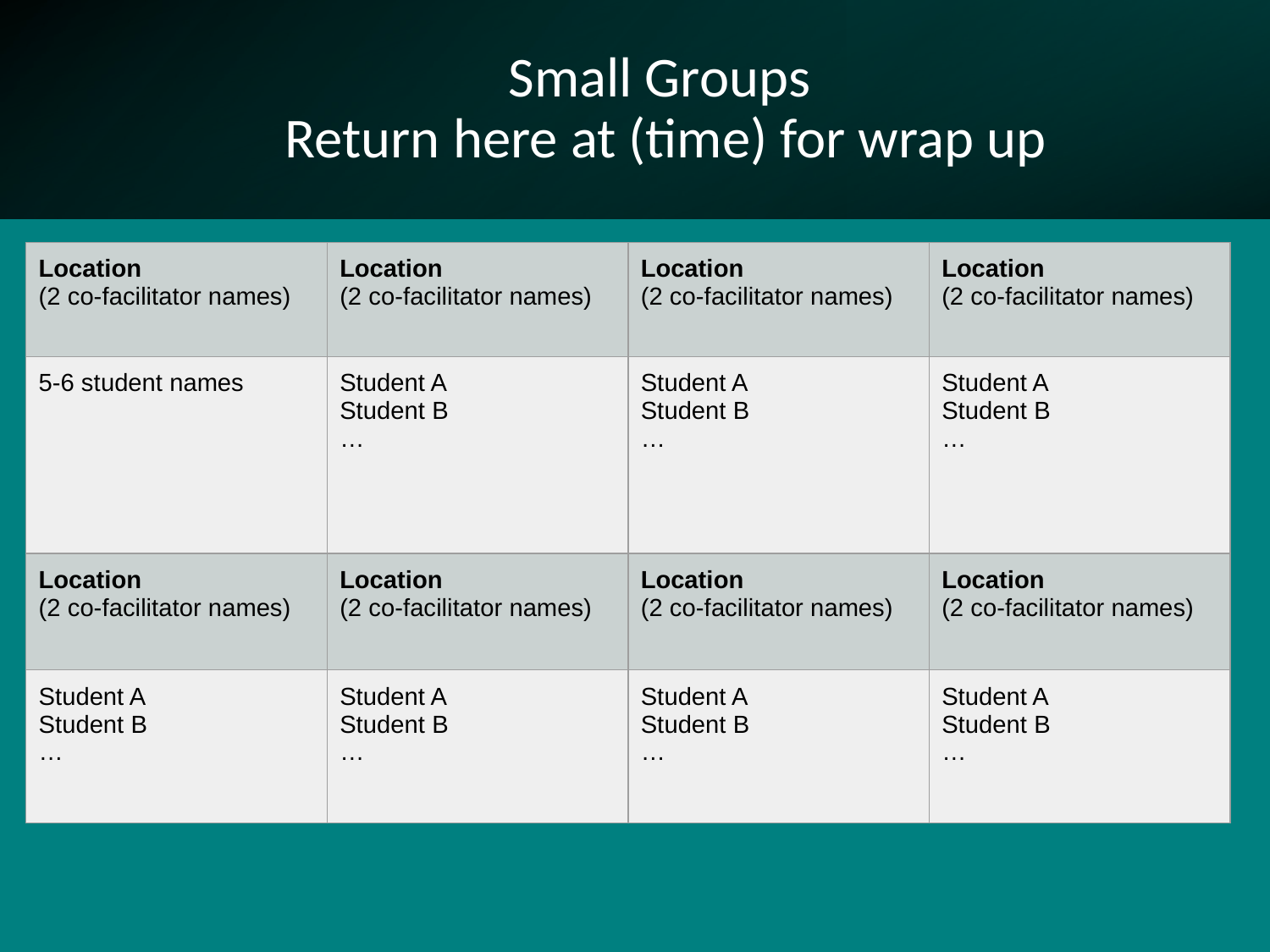

# Small Groups Return here at (time) for wrap up
| Location (2 co-facilitator names) | Location (2 co-facilitator names) | Location (2 co-facilitator names) | Location (2 co-facilitator names) |
| --- | --- | --- | --- |
| 5-6 student names | Student A Student B … | Student A Student B … | Student A Student B … |
| Location (2 co-facilitator names) | Location (2 co-facilitator names) | Location (2 co-facilitator names) | Location (2 co-facilitator names) |
| Student A Student B … | Student A Student B … | Student A Student B … | Student A Student B … |

## Slide 26
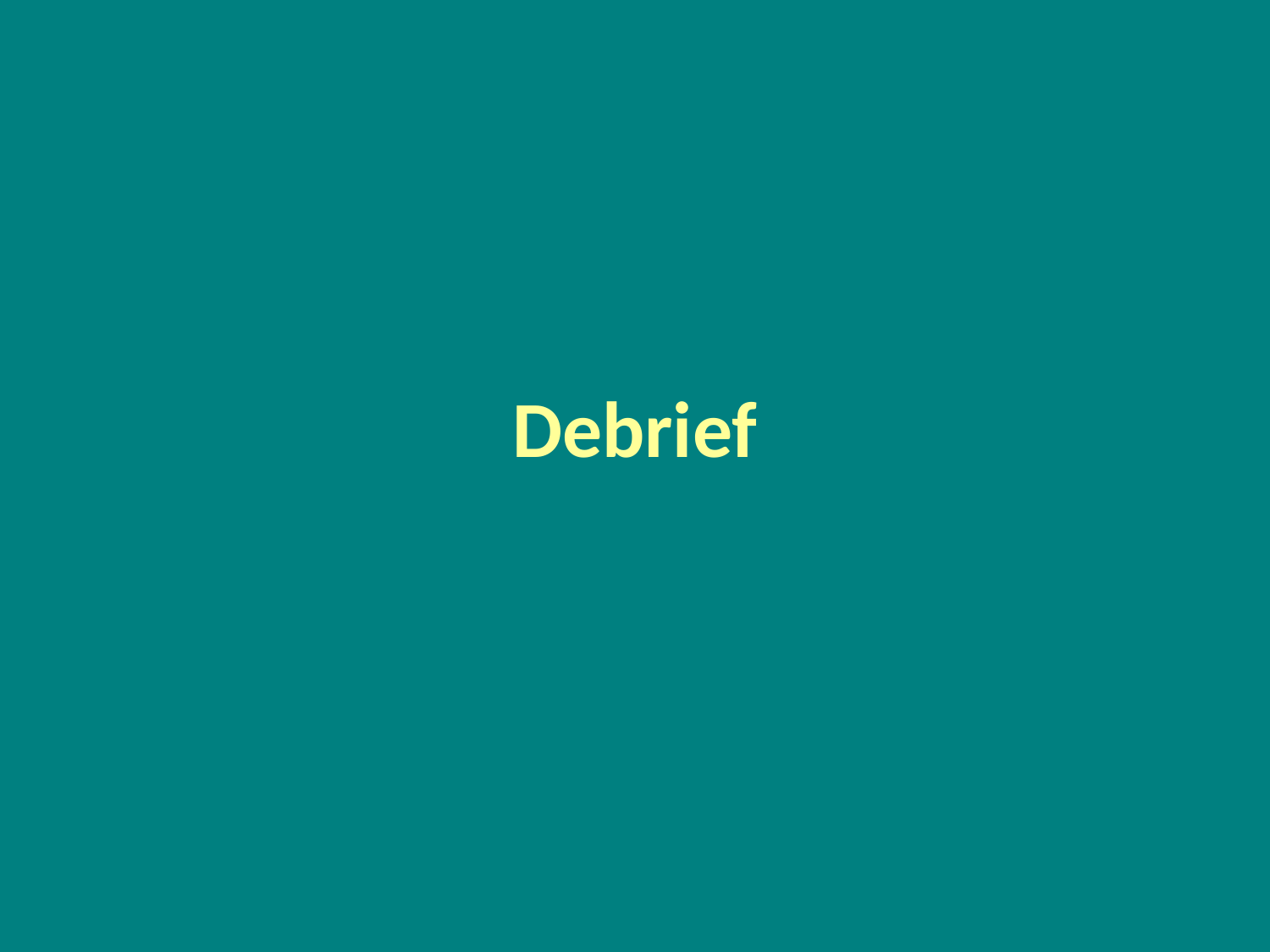

# Debrief

## Slide 27
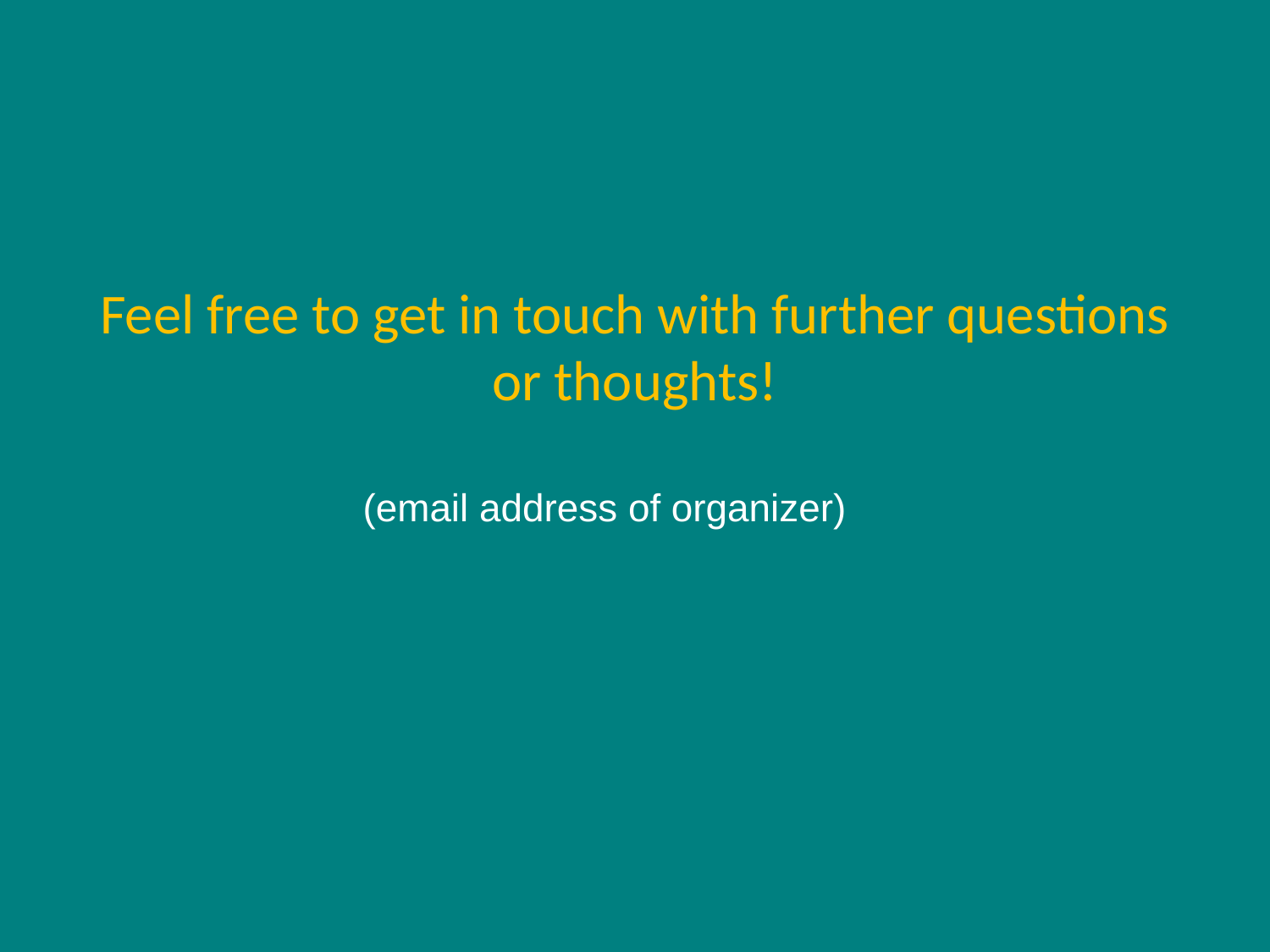

# Feel free to get in touch with further questions or thoughts!
(email address of organizer)
