# Supplementary material for: What to Say When It Matters: Communication Skills to Address Implicit Bias Workshop
Source: MedEdPORTAL. 2025 Apr 15;21:11514. doi: 10.15766/mep_2374-8265.11514 (PMC11997152; doi:10.15766/mep_2374-8265.11514)
Supplement: Supplementary file 1 — Description of Microaggressions Workshop.docxEmail Advertisement.docxSurvey.docxCofacilitator Guide.docxLarge-Group Presentation.pptxGender Bias Simulation.mp4Student in Wheelchair Simulation.mp4Nursing Student Simulation.mp4Skills Card.docxMicroaggression Examples.docx [file mep_2374-8265.11514-s001.zip › I. Skills Card.docx]

***Communication Skills to Address Subtle Acts of Exclusion***

| ***Skill*** | ***Sample Language*** |
| --- | --- |
| ***Pause*** | *"Wow, that didn't feel right. Can we talk about this later? "* |
| ***Interrupt and***  ***ask a question*** | *"Hang on, I'm not sure I heard you correctly. Did you say ...?”* |
| ***Describe***  ***effect using***  ***“I” statements*** | *“I felt my chest tighten when you said x. I need some time to reflect before I discuss this further.”*  *"Whoa... I don't agree with what you just said."* |
| ***Challenge***  ***stereotype***  ***and set***  ***boundaries*** | *“I don’t think that is funny. We don’t perpetuate stereotypes here.”*  *“We don’t tolerate negative comments about (insert identity here). I’d like you to stop.”* |
| ***Make invisible***  ***visible*** | *“I just saw your body language change (describe change) and I felt like I was being dismissed. Did I read that correctly?”* |
| ***Consider Impact*** | *"I don't think that X would be comfortable working with us if they hear that kind of comment."* |
| ***Assume***  ***good intent and educate*** | *“I think you mean to be curious when you ask where I’m from, but that makes me feel like I don’t belong.”* |
| ***If needed,***  ***file a***  ***complaint*** | *Insert school specific mistreatment website/contact information.* |

***Remember that your words convey only part of the message - your tone, pace and non-verbal communication are also extremely important!***

***Allyship Skills***

| ***Skill*** | ***Sample Language*** |
| --- | --- |
| ***Prepare*** | *As team leader, ideally say this when you begin work with a new team:*  *“Sometimes it feels safer if I, as the head of our team, respond to statements of bias we may hear. But I want to empower you to speak up first if you prefer.”* |
| ***Empathize*** | *“I would love to hear more about your experiences of bias if you feel comfortable sharing them.”* |
| ***Distinguish***  ***intent from***  ***impact*** | *“It sounds like you (source of microaggression) didn’t realize X is a valued member of our team when you made that comment.”* |
| ***Educate***  ***and affirm*** | *“We are extremely fortunate to have X on our team. They are a highly trained medical professional and should be treated with respect.”* |
| ***Debrief: Use***  ***I statements,***  ***support and***  ***attend to***  ***emotions*** | *“I was really disappointed witnessing that interaction. I want to make sure I respect your needs - would you like me to say something?... If not, how can I best support you?”*  *“I’d like to take some time to reflect on how that experience was for each of us and to support you.”* |
| ***Affirm and***  ***appreciate*** | *“I recognize that you may feel as though your hard work and intelligence are being overlooked. I want you to know that I see you working really hard, and I know you can be successful in this school.”* |
| ***Reframe*** | *With patient: “I’d like to focus on your health needs now.”*  *With team member: “Is there another way to look at this...?”* |
