# Supplementary material for: What to Say When It Matters: Communication Skills to Address Implicit Bias Workshop
Source: MedEdPORTAL. 2025 Apr 15;21:11514. doi: 10.15766/mep_2374-8265.11514 (PMC11997152; doi:10.15766/mep_2374-8265.11514)
Supplement: Supplementary file 1 — Description of Microaggressions Workshop.docxEmail Advertisement.docxSurvey.docxCofacilitator Guide.docxLarge-Group Presentation.pptxGender Bias Simulation.mp4Student in Wheelchair Simulation.mp4Nursing Student Simulation.mp4Skills Card.docxMicroaggression Examples.docx [file mep_2374-8265.11514-s001.zip › J. Microaggression Examples.docx]

**Microaggression Examples**

- Being called names like ‘honey’ ‘sweetheart’ ‘darling.’ Comments made about how I look or dress. Some men probably assume they’re complimenting me….
- Every time your attending asks a new patient about their sexual partners, he poses the question asking if the patient “had a girlfriend” or “a wife or kids” which made me (gay student) feel uncomfortable and unseen.
- You are a resident finishing up examining a patient when the student on your team comes in to ask you a question. She introduces herself to the patient, “Hi, my name is Maria and I’m the student on the team.” The patient immediately asks the student to empty her wastebasket and requests a fresh towel and then away. The student leaves feeling confused. As you are leaving the room, you overhear the patient ringing her call bell and requesting that “the Mexican housekeeper not come to her room again.”
- In the team room, your resident complains about one of your diabetic patients who is obese. The resident says “Wow, is he disgusting! Why can’t he just get out of bed and start moving?!”
- An attending in clinic, referring to a transgender patient in transition that you just saw together: “That was weird, wasn’t it? What am I supposed to say, hello ma’am or hello sir. I just don’t understand all this nonsense!”
- A male attending or resident gives feedback to a female-identifying medical student that is vague and non-specific. He tells her she is sometimes “poised” and “friendly with patients,” but needs to “take up more space in clinical discussions” and “generally improve on her skills and knowledge.” Her male classmates are given much more tactical and constructive comments about their physical exam skills and clinical reasoning from this attending/resident.
- A preceptor frequently confuses a medical student with another student with a shared racial identity. The student has corrected the preceptor multiple times, but the attending continues to mistake the students and minimizes the error.
- You’re in the OR with the surgeon and a sedated patient on the operating table. The surgeon makes a comment about how the number of tattoos patients have is correlated with how poor their health decision making is.
- You just finished a domestic violence screen, patient history, and physical exam on a pregnant patient. There is no indication that the patient feels unsafe or that she could be facing domestic violence. The physician, upon learning she is from Yemen, makes an assumption that she is an “oppressed” woman and is probably depressed because she has a “hard home life with potentially abusive household.”
- At the end of a clinic visit a patient says to the medical student, “hasta luego, amigo” in an American accent. The student presents as brown. The patient presents as white. The entire clinic visit was completed in English without discussion of either person’s ethnicity, other languages spoken, or identity.
